# Interleukin-1β, lipocalin 2 and nitric oxide synthase 2 are mechano-responsive mediators of mouse and human endothelial cell-osteoblast crosstalk

**DOI:** 10.1038/srep29880

**Published:** 2016-07-19

**Authors:** Vimal Veeriah, Angelo Zanniti, Riccardo Paone, Suvro Chatterjee, Nadia Rucci, Anna Teti, Mattia Capulli

**Affiliations:** 1 Department of Biotechnological and Applied Clinical Sciences, University of L’Aquila, via Vetoio – Coppito 2, 67100 L’ Aquila, Italy; 2 Vascular Biology Laboratory, Anna University K.B. Chandrashekar Research Centre, Chrompet. Chennai (Madras), 600 044, Chennai, India

## Abstract

Endothelial cells are spatially close to osteoblasts and regulate osteogenesis. Moreover, they are sensitive to mechanical stimuli, therefore we hypothesized that they are implicated in the regulation of bone metabolism during unloading. Conditioned media from endothelial cells (EC-CM) subjected to simulated microgravity (0.08*g* and 0.008*g*) increased osteoblast proliferation and decreased their differentiation compared to unit gravity (1*g*) EC-CM. Microgravity-EC-CM increased the expression of osteoblast *Rankl* and subsequent osteoclastogenesis, and induced the osteoblast de-differentiating factor, Lipocalin 2 (Lcn2), whose downregulation recovered osteoblast activity, decreased *Rankl* expression and reduced osteoclastogenesis. Microgravity*-*EC-CM enhanced osteoblast *NO-Synthase2 (NOS2*) and *CycloOXygenase2 (COX2*) expression. Inhibition of NOS2 or NO signaling reduced osteoblast proliferation and rescued their differentiation. Nuclear translocation of the Lcn2/NOS2 transcription factor, NF-κB, occurred in microgravity-EC-CM-treated osteoblasts and in microgravity-treated endothelial cells, alongside high expression of the NF-κB activator, IL-1β. IL-1β depletion and NF-κB inhibition reduced osteoblast proliferation and rescued differentiation. *Lcn2* and *NOS2* were incremented in *ex vivo* calvarias cultured in microgravity*-*EC-CM, and *in vivo* tibias and calvarias injected with microgravity-EC-CM. Furthermore, tibias of botulin A toxin-treated and tail-suspended mice, which featured unloading and decreased bone mass, showed higher expression of *IL-1β, Lcn2* and *Nos2*, suggesting their pathophysiologic involvement in endothelial cell-osteoblast crosstalk.

Bone is a complex tissue, which includes a variety of cell types cooperating to maintain balanced bone formation and resorption. Bone is largely affected by biomechanical stimuli and its homeostasis depends not only on the crosstalk among bone cells but also on their interactions with bone marrow cells, nerve fibers and blood vessels^1^.

Bone is a highly vascularized tissue, and blood vessels largely contribute to bone physiology. Recent studies demonstrated that endothelial cells and osteoblasts cross-regulate each other during osteogenesis^2^,^3^, integrating their signals for the harmonic growth of the skeleton. The tight relationship between these cells is also observed in pathological conditions, including osteoporosis, in which modifications of blood stream and supply occurs before the onset of the disease^4^. Several factors contribute to the molecular interactions between endothelial cells and osteogenic cells, and in the context of our study, Interleukin (IL)-1β, Lipocalin 2 (LCN2) and the Nitric Oxide (NO) pathways were found to be largely implicated in the pathological crosstalk between vascular endothelium and osteoblasts induced by mechanical failure.

IL-1β is a pro-inflammatory cytokine produced as a biologically inert pro-peptide that requires cleavage by caspase-1 to generate the active cytokine^5^. In bone, it is a pro-osteoclastogenic factor^6^ and its expression is increased by estrogen deficiency, thus contributing to the increase of osteoclast bone resorption in postmenopausal osteoporosis^7^. In contrast, the role of IL-1β in osteogenesis has not yet been elucidated^8^, although it is known that IL-1β increases in response to IL-6, a potent anti-osteogenic factor and inducer of bone resorption^9^.

Lipocalin 2 (LCN2) is a pleiotropic and acute-phase protein that binds iron-loaded siderophores and acts as a bacteriostatic agent^10^. It plays roles in cell proliferation, differentiation and survival, and is up-regulated by inflammation and cancer^11^. Our previous work has demonstrated that LCN2 is the most up-regulated gene in osteoblasts subjected to simulated microgravity and exhibits an important negative effect on bone formation^12^,^13^, suggesting a causative relationship among mechanical unloading, LCN2 induction and osteoblast impairment.

NO is a short-lived gaseous molecule that affects the function of many cells^14^. It is released by endothelial cells in response to relaxation stimuli and impairs the contraction of vascular smooth muscle cells, with the final goal to reduce the peripheral blood pressure^15^. The NO signal relies on the activity of various Nitric Oxide Synthase (NOS) isoforms^16^, among which NOS2 represents an important endothelial inducible enzyme also expressed by bone cells^17^. NO has an important role in bone formation^18^, and NOS2 is activated by IL-1β in bone cells, contributing to inflammation-induced bone loss^18^.

The detrimental role of low mechanical strength on bone metabolism is well known and several pathways have been recognized so far to contribute to bone loss in this condition^19^. In contrast, very little is known on the role played by endothelial cells in unloaded bone. In this study, we hypothesized that mechanical forces regulate bone formation and resorption through the endothelial cells and that the mechano-response of endothelial cells to low strength contributes to the reduced osteogenesis and increased bone resorption observed in pathological conditions, such as disuse osteoporosis due to ageing, muscle and bone injuries, paralysis, sarcopenia, muscular dystrophy or prolonged bed-resting. We observed that unloaded endothelial cells release IL-1β, which contributes to the enhancement of the NO signal and the priming of osteoblasts to synthesize LCN2. These molecules are key components of a complex cascade of events, involving NOS2 and the NF-κB pathways, that unbalance bone remodeling and cause bone loss as confirmed in *in vivo* mouse models of mechanical unloading, which recapitulate the features of human osteoporosis induced by disuse.

## Results

### Role of endothelial cells on osteogenesis and osteoclastogenesis in response to decreased mechanical forces

**Figure 1:**
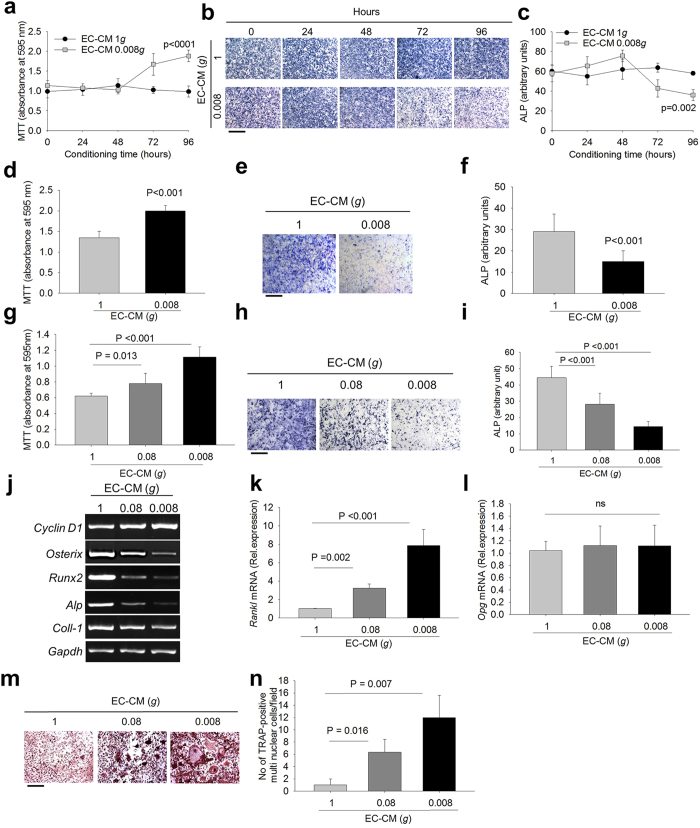
Effects of Endothelial Cell Conditioned Medium (EC-CM) on osteoblasts. Primary endothelial cells isolated from 7 week-old CD1 mouse aorta were subjected to unit gravity (1*g*) and microgravity (0.008*g*) on microbeads for the indicated times, then EC-CM were collected and used to treat primary osteoblasts isolated from 7-day old CD1 mouse calvarias for 48 hours. **(a)** MTT proliferation assay. **(b)** Representative images of ALP cytochemical staining. **(c)** Densitometric quantification of ALP staining. The human endothelial cell line (EA.hy926 cells) was subjected for 96 hours to 1*g* and 0.008*g* on microbeads, then the EC-CM were collected and used to treat mouse primary osteoblasts for 48 hours. **(d)** MTT proliferation assay. **(e)** Representative images of ALP cytochemical staining. **(f)** Densitometric quantification of ALP staining. Primary endothelial cells isolated from 7-week old CD1 mouse aorta were subjected to 1*g*, 0.08*g* and 0.008*g* on microbeads for 96 hours, then EC-CM were collected and used to treat primary osteoblasts isolated from 7-day old CD1 mouse calvarias for 48 hours. **(g)** MTT proliferation assay. **(h)** Representative images of ALP cytochemical staining. **(i)** Densitometric quantification of ALP staining. **(j)** RT-PCR analysis of mRNA expression of the proliferation marker, *Cyclin D1*, and the osteoblast differentiation markers, *Osterix, Runx2, Alp* and *Collagen type 1 chain-α1 (Coll-1*), normalized versus *Gapdh.* Real time RT-PCR of **(k)** *Rankl* and **(l)** *Opg* mRNA expression in mouse primary osteoblasts treated with 1*g*-, 0.08*g-* and 0.008*g*-EC-CM. Total bone marrow cells were isolated from the long bones of 7-day old CD1 mice and cultured with 1*g-*, 0.08*g-* and 0.008 g-EC-CM. **(m)** Representative images of TRAcP staining and (**n**) quantification of TRAcP-positive multinucleated cells per field. Images are representative and data are the mean ± SD of 3 independent experiments (one way RM ANOVA). Bar = 100 μm. All gels have been run under the same experimental conditions.

Mouse primary endothelial cells isolated from aorta expressed the endothelial markers, VEGF and PECAM, confirming the endothelial enrichment of the cultures (Supplementary Fig. S1a,b). Endothelial cells were loaded on microcarriers (Supplementary Fig. S1c) and exposed to unit gravity (1*g*) or simulated microgravity (0.08*g* and 0.008*g*), using the Rotating Wall Vessel (RWV) bioreactor, developed and approved by the USA National Aeronautics and Space Administration (NASA), as a means to lower the mechanical forces acting on the cells in a condition of low shear stress. Endothelial Cell-Conditioned Media (EC-CM) were collected at different time points of 1*g*, 0.08*g* and 0.008*g* cultures and used to treat primary osteoblasts from 7-day old mouse calvarias. We first analyzed whether the osteoblast cultures incubated in 1*g*-EC-CM exhibited any change of the variables investigated in the study compared to osteoblasts exposed to EC-CM derived by standard endothelial cell cultures. We observed that osteoblast proliferation (Supplementary Fig. S2a), Alkaline Phosphatase (ALP) activity (Supplementary Fig. S2b,c) and osteoblast gene expression (Supplementary Fig. S2d–f) were indistinguishable between the two conditions. Furthermore, standard- and 1*g*-EC-CM did not induce osteoclastogenesis in bone marrow mononuclear cell cultures. In contrast, osteoblasts exhibited higher proliferation and lower differentiation in the presence of 0.008*g*-EC-CM harvested at different time points (0-96 hours) (Fig. 1a–c) compared to 1*g*-EC-CM. Similar results were observed incubating human (Supplementary Fig. S3a–c) or mouse primary osteoblasts (Fig. 1d–f) in 0.008*g*-EC-CM from the human endothelial cell line (EA.hy926), suggesting species cross-reactivity of the implicated pathways. Furthermore, mouse primary osteoblasts cultured in 0.08*g*- and 0.008*g*- mouse EC-CM showed progressive increase of proliferation and decrease of differentiation (Fig. 1g–i) in a manner dependent on intensity of microgravity.

Consistent with the previous results, treatment of osteoblasts with 0.08*g*- and 0.008*g-*EC-CM increased the mRNA expression of the proliferation marker, *Cyclin D1*, and decreased the mRNA expression of the differentiation markers *Osterix, Runx2, ALP* and *Collagen type 1 chain-α1* (Fig. 1j) in mouse osteoblasts incubated with mouse EC-CM. Furthermore, 0.08*g*- and 0.008*g-*EC-CM increased osteoblast *Receptor activator of NF-κB transcription factor Ligand (Rankl*) mRNA expression in a manner dependent on microgravity intensity, with no modulation of *osteoprotegerin (Opg*) (Fig. 1k,l) compared to 1*g*-EC-CM. Reliably, 0.08*g*- and 0.008*g-*EC-CM stimulated osteoclast formation in total bone marrow cell cultures (Fig. 1m,n), but not in purified bone marrow mononuclear cell cultures (Supplementary Fig. S4a,b), indicating that the effect of unloaded endothelial cells on osteoclastogenesis was only mediated by supporting osteoblasts.

### Involvement of LCN2

**Figure 2:**
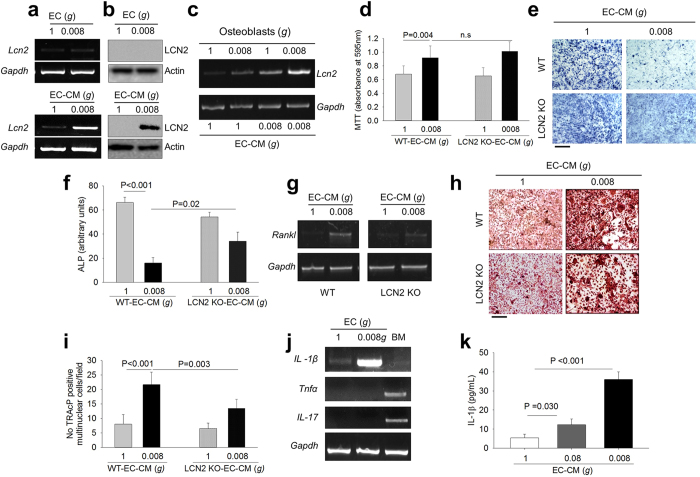
Role of LCN2 in endothelial cell-osteoblast crosstalk. **(a)** RT-PCR of *Lcn2* in 1*g*- and 0.008*g*-treated mouse primary endothelial cells (upper panels) and mouse primary osteoblasts (lower panels), normalized versus *Gapdh.* **(b)** Western blot analysis of LCN2 protein in 1*g*- and 0.008*g*-treated mouse primary endothelial cells (upper panels) and mouse primary osteoblasts (lower panels) treated with 1*g-* and 0.008*g-*EC-CM, normalized versus actin. **(c)** RT-PCR of *Lcn2* in mouse primary osteoblasts subjected to 1*g* and 0.008*g* in the presence of 1*g-* and 0.008*g-*EC-CM, normalized versus *Gapdh.* Wild-type (WT) and LCN2 KO osteoblasts were treated with 1*g*- or 0.008*g*-EC-CM. **(d)** MTT proliferation assay. **(e)** Representative images of ALP cytochemical staining. **(f)** Densitometric quantification of ALP staining. **(g)** RT-PCR of *Rankl* in 1*g-* and 0.008*g-*EC-CM treated mouse primary osteoblasts, isolated from calvarias of WT and LCN2 KO mouse, normalized versus *Gapdh*. Total bone marrow cells were isolated from the long bones of WT and LCN2 KO mice and cultured with 1*g-* or 0.008*g-*EC-CM. **(h)** Representative images of TRAcP staining. **(i)** Quantification of TRAcP positive multinucleated cells per field. **(j)** RT-PCR of the LCN2 upstream activators *IL-1β, Tnfα* and *IL-17* in 1*g-* or 0.008*g-*treated mouse primary endothelial cells, normalized versus *Gapdh*. Bone marrow cells (BM) were analyzed as technical positive control for *Tnfα* and *IL-17.* **(k)** ELISA assay for IL-1β in EC-CM from mouse primary endothelial cells exposed for 96 hours to 1*g*, 0.08*g* or 0.008*g*. Images are representative and data are the mean ± SD of at least 3 independent experiments (Student’s *t* test). All gels have been run under the same experimental conditions. Actin was evaluated on the same blotting of LCN2. Bar = 100 μm.

The effects of 0.08*g*- and 0.008*g-*EC-CM on osteoblasts were similar to the effects induced by the direct exposure of osteoblasts to simulated microgravity, which are mediated by the mechano-responding gene, LCN2^12^,^13^. LCN2 mRNA and protein expression was undetectable in endothelial cells cultured in microgravity, but they increased in 0.008*g-*EC-CM-treated osteoblasts compared to controls (Fig. 2a,b), suggesting that endothelial cells released mediators that stimulated LCN2 production in the osteoblasts. This effect was additive to the increase of *Lcn2* mRNA directly induced in osteoblasts by the low gravitational force (Fig. 2c).

To confirm the role of LCN2 in this context, we treated osteoblasts from LCN2 knockout (KO) and wild-type mice with 0.008*g*-EC-CM and observed that LCN2 deficiency did not affect the 0.008*g-*EC-CM-mediated increase of proliferation (Fig. 2d), but it reduced the impairment of osteoblast differentiation (Fig. 2e,f) and the increase of *Rankl* mRNA expression (Fig. 2g). Consistently, LCN2 deficiency reduced the 0.008*g-*EC-CM-mediated increase of osteoclast formation in total bone marrow cell cultures (Fig. 2h,i).

To identify the upstream molecules released by endothelial cells under microgravity, we focused on TNFα, IL-1β and IL-17, known to be secreted factors that upregulate LCN2 expression in different cell types^20^,^21^. In our experimental conditions, only IL-1β mRNA expression was enhanced in endothelial cells cultured at 0.008*g* versus controls (Fig. 2j), in agreement with a recent report showing that IL-1β is one of the most upregulated secreted protein in endothelial cells during spaceflight^22^. The release of the IL-1β protein was increased in EC-CM in a manner dependent on the intensity of microgravity (Fig. 2k).

### Involvement of NO pathway

Our results showed that LCN2 reduces osteoblast differentiation but does not affect osteoblast proliferation. To investigate the underlying mechanisms, we treated osteoblasts with the cell proliferation inhibitor, hydroxyurea, which harmed the 0.008*g-*EC-CM-mediated increase of osteoblast proliferation (Supplementary Fig. S5a) and *Cyclin D1* overexpression (Supplementary Fig. S5b), and partially blocked the impairment of osteoblast differentiation (Supplementary Fig. S5c,d), indicating that the two events were only partially associated.

**Figure 3:**
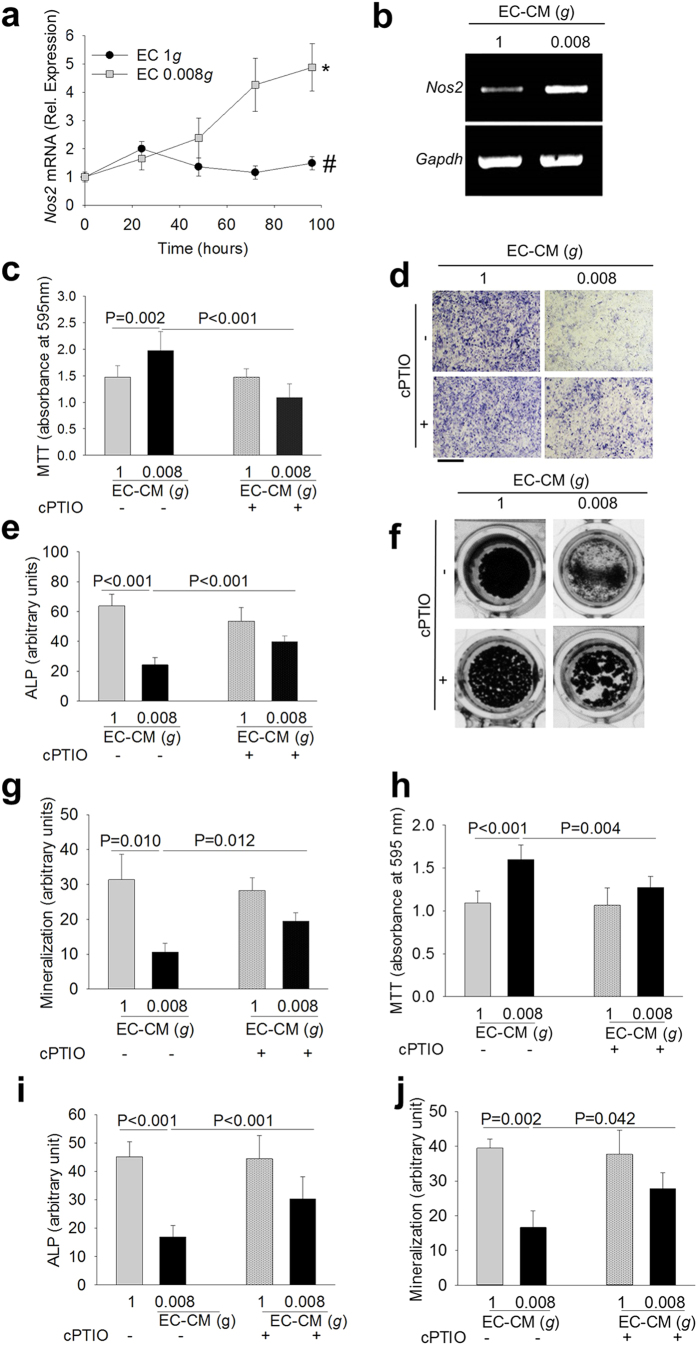
Role of the NOS2-NO-COX2 pathway in endothelial cell-osteoblast crosstalk. Mouse primary endothelial cells were subjected to 1*g* and 0.008*g* for the indicated times. **(a)** Real time RT-PCR analysis of *Nos2* normalized versus *Gapdh*. **(b)** EA.hy976 cells were subjected to 1*g* and 0.008*g* for 96 hours. RT-PCR of *Nos2* normalized versus *Gapdh.* Mouse primary osteoblasts were treated with 1*g-* or 0.008*g-*EC-CM in the absence or presence of the NO quencher cPTIO. **(c)** MTT proliferation assay. **(d)** Representative images of ALP cytochemical staining. **(e)** Densitometric quantification of ALP staining. **(f)** Representative images of mineralized nodule formation by the Von Kossa staining. **(g)** Densitometric quantification of mineralization. Frozen 1*g-* and 0.008*g-*EC-CM were de-frozen in ice and used to treat mouse primary osteoblasts in the absence or presence of the NO quencher, cPTIO. **(h)** MTT proliferation assay. **(i)** Densitometric analysis of ALP cytochemical staining. **(j)** Densitometric quantification of mineralization (Von Kossa staining). Data are the mean ± SD of 3 independent experiments (Student’s t test). Bar = 100 μm. All gels have been run under the same experimental conditions.

Previous studies demonstrated that the reduction of mechanical forces increases NOS2 expression in endothelial cells^23^ and in a variety of other cell types^24^. NOS2 produces more NO than the other NOS isoforms^25^ and NO is one of the important regulators of osteoblast differentiation^26^,^27^,^28^. Moreover, NO has a biphasic action, stimulating osteoblast differentiation at low concentration and inhibiting this process at high concentration^29^,^30^. In our experimental conditions, we observed that *Nos2* mRNA expression was higher after exposure of endothelial cells to low gravity (Fig. 3a,b). In osteoblasts incubated with 0.008*g-*EC-CM along with the NO quencher, 2-4-carboxyphenyl-4,4,5,5-tetramethylimidazoline-1-oxyl-3-oxide (cPTIO), we observed lower 0.008*g*-EC-CM-mediated increase of proliferation (Fig. 3c) and partial rescue of impaired osteoblast differentiation (Fig. 3d,e) and matrix mineralization (Fig. 3f,g).

**Figure 4:**
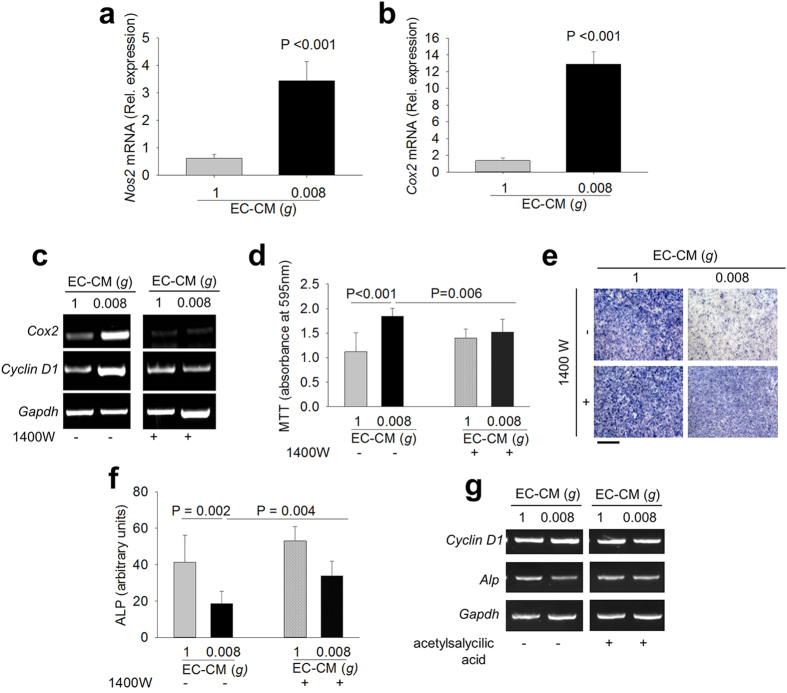
Role of the NOS2-NO-COX2 pathway in endothelial cell-osteoblast crosstalk. Mouse primary osteoblasts were treated with 1*g-* and 0.008*g-*EC-CM. Real time RT-PCR of **(a)** *Nos2* and **(b)** *Cox2* normalized versus *Gapdh*. Mouse primary osteoblasts were treated with 1*g-* or 0.008*g-*EC-CM in the absence or in the presence of the NOS2 inhibitor 1400 W. **(c)** RT-PCR of *Cox2* and *Cyclin D1* normalized versus *Gapdh*. **(d)** MTT proliferation assay. **(e)** Representative images of ALP cytochemical staining. **(f)** Densitometric quantification of ALP staining. Osteoblasts were treated with 1*g-* and 0.008*g-*EC-CM in the absence or presence of the COX inhibitor acetylsalicylic acid. **(g)** RT-PCR analysis of *Cyclin D1 and Alp* normalized versus *Gapdh*. Images are representative and data are the mean ± SD of at least 3 independent experiments (Student’s t test). Bar = 100 μm. All gels have been run under the same experimental conditions.

Interestingly, a serendipity observation revealed that cPTIO counteracted the effects of 0.008*g-*EC-CM also in osteoblasts treated with frozen conditioned medium (Fig. 3h–j). This finding was in overt contradiction with the well-known property of NO, a gaseous molecule with a very short half-life^31^. Therefore, we questioned whether 0.008*g-*EC-CM induced the intracellular NOS2/NO signaling in osteoblasts as well, which could produce their own NO. To address this aspect we evaluated the expression of *Nos2* and *CycloOXygenase 2 (Cox2*) in osteoblasts and observed that 0.008*g*-EC-CM increased both mRNAs in mouse primary osteoblasts compared to control media (Fig. 4a,b). COX2 is a downstream target of NOS2^32^ and a known mitogen^33^, which induces osteoblast proliferation^34^. Of note, inhibition of osteoblast NOS2 by the NOS2 specific antagonist, N-(3-[aminomethyl]benzyl) acetamidine (1400 W), partially prevented the 0.008*g-*EC-CM-induced mRNA expression of *Cox2* and *Cyclin D1* (Fig. 4c). Moreover, inhibition of osteoblast NOS2 or COX2 using 1400 W (Fig. 3d–f) or acetylsalicylic acid (Fig. 4g), respectively, inhibited the 0.008*g-*EC-CM-mediated proliferation and partially rescued osteoblast differentiation. These results indicate that the direct exposure of endothelial cells to reduced mechanical forces, as well as the treatment of osteoblasts with 0.008*g-*EC-CM, induced NOS2/NO signal in both cell types.

### Involvement of NF-ĸB

**Figure 5:**
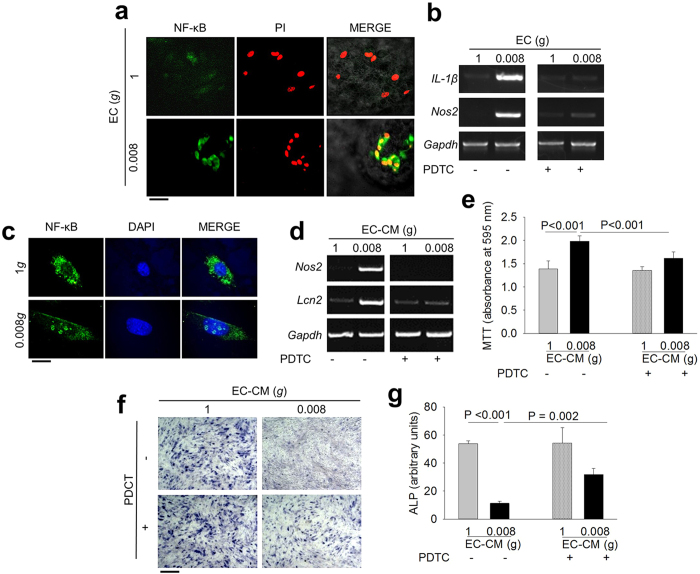
Role of NF-κB in endothelial cell-osteoblast crosstalk. **(a)** Immunofluorescence analysis of p65 NF-ĸB subunit subcellular localization in 1*g-* and 0.008*g*-treated mouse primary endothelial cells. **(b)** RT-PCR analysis of *IL-1β* and *Nos2* in mouse primary endothelial cells subjected to 1*g* and 0.008*g* in the absence or presence of the NF-κB inhibitor PDTC, normalized versus *Gapdh*. **(c)** Immunofluorescence analysis of p65 NF-ĸB subunit subcellular localization in 1*g-* and 0.008*g-*EC-CM treated mouse primary osteoblasts. Osteoblasts were incubated in 1*g-* or 0.008*g-*EC-CM in the absence or presence of PDTC. **(d)** RT-PCR of *Nos2* and *Lcn2* normalized versus *Gapdh*. **(e)** MTT proliferation assay. **(f)** Representative images of ALP cytochemical staining. **(g)** Densitometric quantification of ALP staining. Images are representative and data are the mean ± SD of at least 3 independent experiments (Student’s *t* test). Bar = (**a**) 30 μm; (**c**) 10 μm; (**f**) 100 μm. All gels have been run under the same experimental conditions.

IL-1β and NOS2 are transcriptionally induced by the NF-ĸB transcription factor^35^,^36^. Moreover, IL-1β requires NF-ĸB activation to induce LCN2 expression^37^. In our experimental conditions, immunofluorescence analysis demonstrated that 0.008*g* activated NF-ĸB in endothelial cells inducing its nuclear translocation (Fig. 5a). Therefore, we blocked this NF-ĸB activation during the exposure of endothelial cells to simulated microgravity using the specific inhibitor, pyrroledine dithiocarbamate (PDTC), and observed no overexpression of *Nos2* and *IL-1β* mRNAs (Fig. 5b).

NF-ĸB nuclear translocation occurred also in 0.008*g-*EC-CM-treated osteoblasts (Fig. 5c). Furthermore, 0.008*g-*EC-CM failed to induce overexpression of the osteoblast NF-ĸB target genes, *Nos2* and *Lcn2*, in the presence of PDTC (Fig. 5d). Consequently, in this circumstance, 0.008*g-*EC-CM did not induce proliferation and partially prevented the reduction of osteoblast differentiation (Fig. 5e–g).

### Role of IL-1β in the osteoblast NF-ĸB/LCN2/NOS2 pathways

**Figure 6:**
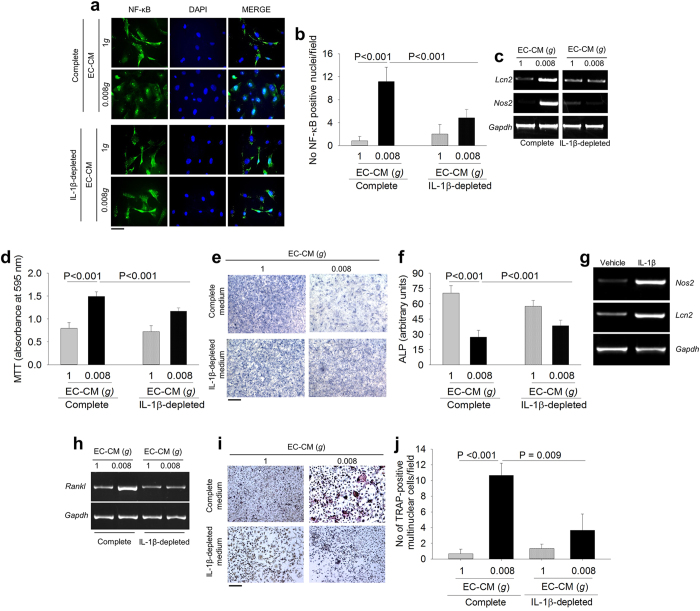
Effect of IL-1β depletion on endothelial cell-osteoblast crosstalk. Mouse primary osteoblasts were treated with complete or IL-1β-depleted media as indicated. **(a)** Immunofluorescence analysis of p65 NF-ĸB subunit subcellular localization. **(b)** Quantification of p65 NF-ĸB-positive nuclei per field. **(c)** RT-PCR of *Lcn2 and Nos2* normalized versus *Gapdh*. **(d)** MTT assay to analyze cell proliferation. **(e)** Representative images of ALP cytochemical staining. **(f)** Densitometric quantification of ALP activity. **(g)** RT-PCR of *Lcn2 and Nos2* normalized versus *Gapdh* in mouse primary osteoblasts treated with vehicle (PBS) or mouse IL-1β recombinant protein. **(h)** RT-PCR of *Rankl* normalized versus *Gapdh* in mouse primary osteoblasts treated with complete or IL-1β-depleted media. Total bone marrow cells were isolated from the long bones of WT mice and treated with complete or IL-1β-depleted 1*g*- or 0.008*g*-EC-CM. **(i)** TRAcP cytochemical staining. **(j)** Quantification of TRAcP-positive multinucleated cells. Images are representative and data are the mean ± SD of 3 independent experiments (Student’s t test). Bar = (**a**) 20 μm; (**e**) 100 μm; (**j**) 50 μm. All gels have been run under the same experimental conditions.

Given that IL-1β is a known inducer of LCN2 and NOS2 through the activation of NF-ĸB^38^,^39^, we asked whether NF-ĸB nuclear translocation was triggered in osteoblasts by the IL-1β released in the 0.008*g-*EC-CM. Results demonstrated that IL-1β depletion by anti-IL-1β antibody prevented the 0.008*g-*EC-CM-mediated NF-ĸB nuclear translocation (Fig. 6a,b). Consequently, IL-1β depleted 0.008*g-*EC-CM failed to induce LCN2 and NOS2 overexpression in osteoblasts (Fig. 6c). Furthermore, IL-1β depletion reduced the 0.008*g-*EC-CM mediated proliferation and partially rescued osteoblast differentiation (Fig. 6d–f). Consistently, treatment for 24 hours with 4 ng/ml recombinant mouse IL-1β induced the transcriptional expression of *Nos2* and *Lcn2* in osteoblasts (Fig. 6g). Intriguingly, RT-PCR analyses revealed that osteoblast *Lcn2* and *Nos2* were independently activated by IL-1β through the NF-ĸB nuclear translocation. In fact, *Nos2* was up-regulated by 0.008*g-*EC-CM also in LCN2 KO osteoblasts, while the NOS2 inhibitor, 1400 W, impaired the induction of *Cox2* by 0.008*g-*EC-CM, but did not affect *Lcn2* expression (Supplementary Fig. S6). Finally, IL-1β depletion mitigated the effect of 0.008*g-*EC-CM on osteoblast *Rankl* expression (Fig. 6h) and on osteoclastogenesis in total bone marrow cell culture (Fig. 6i,j).

### Involvement of IL-1β/LCN2/NOS2 signals in *ex vivo* and *in vivo* bone

**Figure 7:**
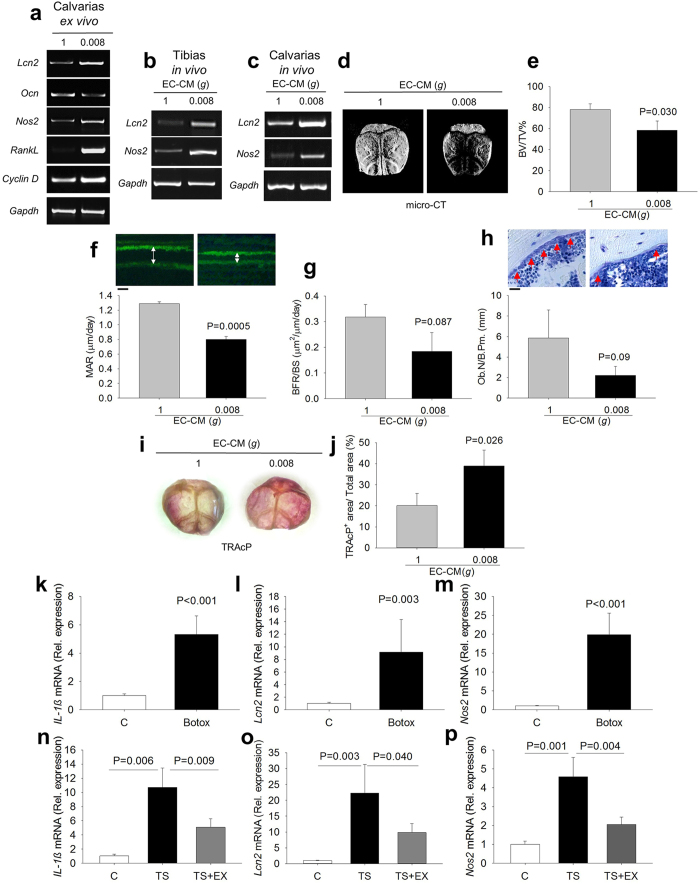
*Ex vivo* and *in vivo* activation of the IL-1β/NOS2/LCN2 pathway. **(a)** Calvarias were isolated from 7-day old C57BL/6J mice and cultured *ex vivo* with 1*g-* or 0.008*g-*EC-CM. RT-PCR of *Lcn2, Ocn, RankL, Nos2,* and *Cyclin D1* normalized versus *Gapdh*. 1*g-* and 0.008- EC-CM were injected in 4 week-old C57BL/6J mice **(b)** into tibias (intra-tibial injection) and **(c)** onto calvarias (subcutaneous injection). RT-PCR of *Nos2* and *Lcn2* normalized versus *Gapdh*. **(d)** Representative of micro-CT images of 4 week-old C57BL/6J mouse calvarias treated *in vivo* with 1*g-* and 0.008*g-*EC-CM. **(e)** Quantitative analysis of calvarial bone volume over total tissue volume (BV/TV). **(f)** Calcein labeling (green fluorescence, double white arrows) (upper panels) and quantification of Mineral Apposition Rate (MAR) (lower panel). **(g)** Quantification of Bone Formation Rate (BFR). **(h)** Histological images of toluidine blue stained histological sections (osteoblasts, red arrows) (upper panels) and quantification of osteoblast number over bone perimeter (Ob.N./B.Pm.) (lower panel), **(i)** Representative images of whole mount TRAcP staining and **(j)** TRAcP quantification. Eight-week old C57BL/6J mice were subjected to Botox injection as described in methods, RNA was isolated from their long bones and mRNA expression of **(k)** *IL-1β*, **(l)** *Lcn2* and **(m)** *Nos2* was analyzed by real-time RT-PCR. Eight-week old C57BL/6J mice were subjected to hind limb tail suspension (TS), with or without physical exercise (TS + EX) by the forced swim test to counteract the mechanical unloading. Total RNA was isolated from femurs and subjected to real time RT-PCR for **(n)** *IL-1β*, **(o)** *Lcn2* and **(p)** *Nos2*. Images are representative and data are the mean ± SD of at least 3 independent experiments or 3 mice/group (Student’s *t* test). Bar = (**f**) 2 μm; (**h**) 20 μm. All gels have been run under the same experimental conditions.

To investigate whether the regulation of NOS2 and LCN2 pathways could have a pathophysiologic role in the bone tissue, we isolated calvarias from 7-day old wild-type mice and performed *ex vivo* organ culture in the presence of 0.008*g-*EC-CM. This treatment increased the mRNA expression of *Lcn2, Rankl, Nos2* and *Cyclin D1*, and decreased the expression of osteocalcin (*Ocn*) compared to control EC-CM (Fig. 7a). Furthermore, we performed *in vivo* injection of 1*g-* and 0.008*g-*EC-CM onto calvarias and into tibias, respectively (Fig. 7b,c). 0.008*g-*EC-CM increased the mRNA expression of *Lcn2* and *Nos2* in both circumstances and reduced the bone volume over the total tissue volume (Fig. 7e). Consistently, *in vivo* double calcein labeling showed a significant decrease of mineral apposition rate (Fig. 7f) and a trend of decrease of bone formation rate (Fig. 7g) and osteoblast number over bone perimeter (Fig. 7h). Furthermore, the treatment induced an increase of osteoclast-mediated osteolysis, assessed by whole mount TRAcP histochemical staining of the calvarial bones (Fig. 7i,j).

Finally, we investigated the IL-1β, NOS2 and LCN2 pathways in unloaded mouse models obtained by intramuscular injection of botulin toxin in hind limbs, or by tail suspension for 21 days, which are known to induce marked bone loss^13^. The transcriptional expression of these molecules increased in the posterior long bones of both models (Fig. 7k–p). Interestingly, and in agreement with our hypothesis, their expression returned towards normal values in tail suspended mice subjected to physical exercise (Fig. 7n–p), confirming the mechano-responding properties and the pathophysiologic relevance of the described pathways.

## Discussion

The results obtained in our *in vitro, ex vivo* and *in vivo* studies support the involvement of endothelial cells in the response of bone to reduced mechanical forces, which are known to cause severe bone loss and increased risk of fracture. We demonstrated that a tight network of stimuli from endothelial cells and osteoblasts integrate the response of bone to the lowering of physical strengths, suggesting their involvement in the development of disuse osteoporosis. How endothelial cells sense gravitational forces is a matter of speculation. Changes in mechanical strengths are believed to be sensed by adhesion receptors, including selectins, cadherins and integrins, through which the cell undergoes variations in tensions that modify its architecture^40^. This phenomenon reminds the structural principle of “biotensegrity”^41^ and it is thought to cause reorganization and stabilization of the cytoskeletal components, including the nuclear scaffold that, through mechanisms yet to be determined, modulates gene expression^42^.

**Figure 8:**
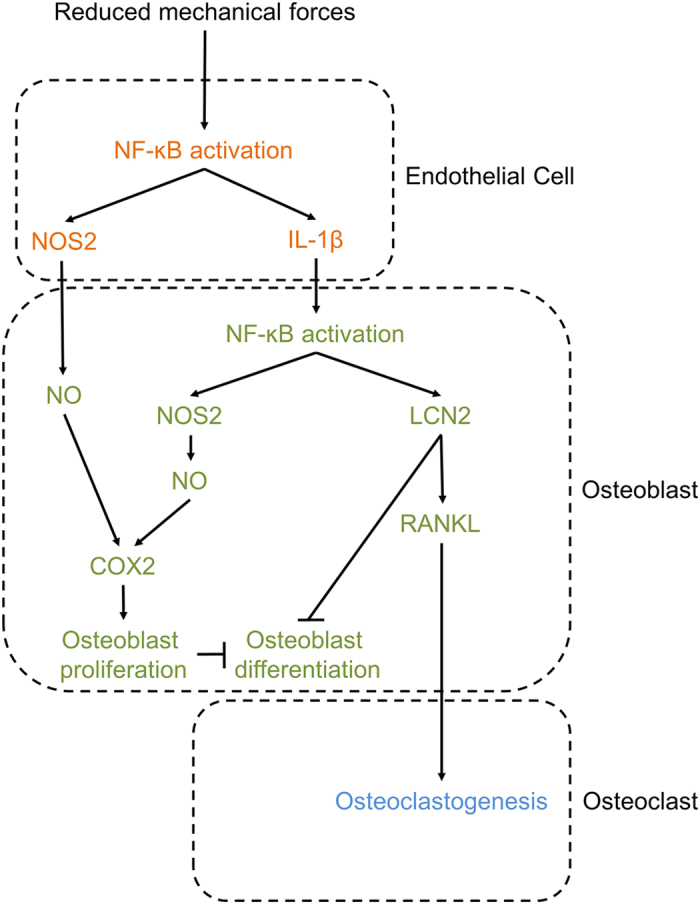
Pathways involved in endothelial cell-osteoblast crosstalk in unloading conditions. Schematic representation of the identified molecular mechanisms. The reduction of mechanical loading activates NF-ĸB nuclear translocation and up-regulates its target genes NOS2 and IL-1β in endothelial cells. Increase of NOS2-NO production enhances proliferation and decreases differentiation in osteoblasts. IL-1β secretion from endothelial cells activates NF-ĸB nuclear translocation in osteoblasts and induces the overexpression of its target genes NOS2 and LCN2. NOS2, through its downstream target COX2, increases proliferation and decreases differentiation in osteoblasts. LCN2 overexpression impairs osteoblast differentiation and induces osteoclastogenesis by increasing osteoblast RANKL expression.

Disuse osteoporosis represents a pathologic condition with high socio-economic burden, whose incidence is steadily increasing in Western countries due to the extended life expectancy. It is secondary to several diseases, including sarcopenia, paralysis, muscular dystrophies, ageing, bone and muscle injuries or prolonged bed-binding due to other disorders^43^,^44^. In this work, we identified the molecules responsible for the endothelial-osteoblast crosstalk in response to decreased mechanical stimuli, and described the network of regulations that finely orchestrates the adaptive response of bone to unloading (Fig. 8). Our results are strengthen by the *in vivo* experiments that fully recapitulated the *in vitro* observations, providing a robust translational relevance to the study. Three of these molecules, IL-1β, NO and LCN2, are soluble factors whose targeting could be exploited for therapy. Notably, systemic treatment with NO inhibitors, like tilarginine acetate, is already approved for clinical trials^45^, and an IL-1β blocker (canakinumab®) is used to combat autoimmune and other diseases^46^,^47^,^48^. We believe that our study provides a rational for extending the employment of these therapies for the treatment of disuse osteoporosis and open a new direction to prevent the deleterious effects of bone unloading. Furthermore, the upregulation of osteoblast LCN2 in response to 0.08 and 0.008*g*-EC-CM confirms the central role for this molecule in the response of osteoblasts to reduced mechanical forces recently demonstrated by our group^12^,^13^, further strengthened by the additive effect of the 0.008*g*-EC-CM over the LCN2 expression directly enhanced in osteoblasts by the microgravity itself. Taken together, the observations of our study suggest that not only LCN2 is directly induced by the reduction of mechanical loading in osteoblasts^12^,^13^, but also that this effect is further potentiated by the unloaded endothelial cells through the IL-1β mediated signal. Although LCN2 is a pleiotropic molecule with a variety of roles in different tissues and conditions, its deletion in mice causes only a mild increase of infection susceptibility^49^, thus supporting the possibility that LCN2 blocking treatment could be developed for therapy in humans.

Our results show that endothelial cells and osteoblasts share the same key transcription factor, NF-κB, to orchestrate the response to unloading. In endothelial cells, NF-κB is directly activated by the low mechanical strengths and induces NOS2 and IL-1β expression, while in osteoblasts it is activated by the IL-1β released by the unloaded endothelial cells and induces NOS2 and LCN2 transcription. Both pathways affect osteoblast activity, NOS2 increasing proliferation and LCN2 impairing differentiation, with the endpoint of reduced matrix nodule formation and mineralization. This effect itself can cause bone loss as a consequence of insufficient osteogenesis. On the other hand, LCN2 also stimulates RANKL production by osteoblasts, which, even in the absence of modulation of OPG, causes the RANKL/OPG ratio to favor osteoclastogenesis and, consequently, bone resorption, thus adding deleterious effects on bone mass. It is clear though that mechanical forces contribute to the balance of bone remodeling also through the endothelial cells, and that deregulation of this delicate integrated signaling network represents one of the biological basis by which osteogenesis is impaired and osteoclastogenesis is enhanced, unbalancing bone turnover.

The study presents some limitations. Simulating microgravity conditions on Earth is not an easy task. We employed the RWV bioreactor, which represents one of the most reliable means to model this situation. The bioreactor has been developed by the NASA and allows tridimensional cultures of adhesive cells through their interaction with microbeads. Our 1*g* cultures were performed incubating endothelial cells on microbeads in non-adhesive Petri dishes, which represent the culture conditions closest, but not identical, to those created by the RWV bioreactor. These tridimensional cultures were indistinguishable from standard bidimensional endothelial cell cultures in terms of the variables investigated in this study. According to our previous work^13^, in key experiments performed with the RWV bioreactor we subjected the endothelial cells to 0.08*g* and 0.008*g*. The experimental conditions between these two settings were identical except for the intensity of the gravitational forces. We observed that the magnitude of the changes observed in our experiments was proportional to the reduction of the gravitational force applied, making us confident that we were indeed observing changes induced by microgravity and not by other factors. Furthermore, some of our findings, for instance the increase of IL-1β in endothelial cells subjected to microgravity, were observed also in endothelial cells in spaceflight^22^, supporting the reliability of our results.

In conclusion, in this study integrating signals have been demonstrated to orchestrate the response of bone to low mechanical strengths and to contribute to the bone loss observed in disuse osteoporosis. These signals associate the response of endothelial cells to the response of osteoblasts, strengthening the concept of the importance of the molecular cooperation between these two cell types to keep the bone tissue healthy.

## Materials and Methods

Materials and conventional methods are described in Supplementary Materials and Methods. Uncropped PCRs and Western blots for Figs 1j, 2a,f,i, 5d and 7c are shown is Supplementary Figs S7–S11.

### Ethics and animal studies

Procedures involving animals and their care were conducted in accordance with national and international laws and policies (European Economic Community Council Directive 86/609, OJ L 358, 1, December 12, 1987; Italian Legislative Decree 4.03.2014, n.26, Gazzetta Ufficiale della Repubblica Italiana no. 61, March 4, 2014) and received Institutional approval by the Internal Ethical Board regulation (University of L’Aquila, Italy) headed by Prof. Edoardo Alesse. Animals were purchased from Charles River (Milan, Italy). Animal studies were conducted in compliance with the ARRIVE requirements and are described in Supplementary Table S1.

### Rotating wall vessel (RWV) bioreactor

The RWV Bioreactor-model STLV, size 55 ml (Synthecon CELLON S.ar.l, Strassen, Luxembourg) was used for this study; it was developed and approved by the NASA for *in vitro* reduced mechanical forces research. This is a bubble-free culture vessel containing membrane diffusion gas exchange, where the cells on microbeads and culture medium rotate inside the vessel. Microbeads are used as a solid body for cells in suspension, especially if they are adhesive cells like endothelial cells. Microbeads and cells rotate inside the vessel of the bioreactor and this horizontal rotation gives cells a constant randomization of the normal gravity vector, thus mimicking a state of free fall, which simulate aspects of microgravity. Rotation about a horizontal axis reduces shear and turbulence compared to conventional stirred bioreactors. Information is available at http://www.biocompare.com/Product-Reviews/41543-Rotary-Cell-Culture-System-RCCS-8DQ-From-Synthecon-Inc/.

### Processing of endothelial cells for exposure to reduced mechanical forces

Mouse primary endothelial cells or EA.hy926 cells were collected after confluence and re-suspended in DMEM-10% FBS containing CultiSpher-G^®^ microbeads (1 g/L medium), at a cellular density of 1.2 × 10^6^ cells/ml, to allow cell-cell and cell-substrate interaction on microbeads. This suspension was grown for up to 96 hours into the RWV bioreactor under a 50- or 16-rpm rotations, which lead to a condition of 0.08*g* and 0.008*g*, respectively, or in non-adhesive Petri dishes for the unit gravity condition (1*g*). We used standard bidimensional endothelial cultures (without microbeads) to check any change induced by the 1*g* tridimensional cultures (with microbeads) on the variables investigated in the study. For some experiments, cultures were performed in the presence or absence of PDTC (500 μM). At the end of the experiment, cells were collected, along with microbeads and conditioned medium, and separated by centrifuge. Endothelial cells were processed for RNA and protein extraction or fixed for immunofluorescence experiments. EC-CM were collected on ice and immediately frozen at −80 °C. For some NO signaling experiments, conditioned media were used without freezing, as indicated in the results section.

### Treatment with conditioned media

1*g*-, 0.08*g*- and 0.008*g*-EC-CM were used to: i) incubate *in vitro* osteoblasts, total bone marrow cells and purified bone marrow mononuclear cells, ii) incubate *ex vivo* calvaria cultures and iii) inject *in vivo* intra-tibially, or subcutaneously in the area above the calvarial bones. In some *in vitro* experiments, osteoblasts were treated with EC-CM along with 1400 W (10 μM), PDTC (500 μM), acetylsalicylic acid (1 mM), cPTIO (100 μM) and hydroxyurea (200 μM).

### ELISA assay and IL-1β depletion

IL-1β present in the conditioned media was quantified by ELISA method using Ray Bio^®^ mouse IL-1β ELISA kit protocol (Cat# ELM-IL1beta-001), according to the manufacturer instructions. We used the same kit also to deplete IL-1β from condition media. For this purpose, we incubated 1*g-* and 0.008*g-*EC-CM in ELISA wells with immobilized IL-1β antibody for 1 hour at room temperature. Then condition media were removed and used to treat osteoblasts. The stripes were further processed for ELISA to confirm the depletion of IL-1β. Control media were kept in uncoated wells in the same conditions.

### *Ex vivo* calvarial culture

Calvarias were dissected from 8-week old C57BL/6J mice, cleaned thoroughly from soft tissues and cultured with 1*g* and 0.008*g* EC-CM. After 48 hours, calvarias were collected and processed for RNA extraction using TRIzol method.

### *In vivo* intra-tibial and subcutaneous calvarial injection

Eight-week-old C57BL/6J mice were anesthetized and 1*g* or 0.008*g* EC-CM were injected locally into the tibia or subcutaneously onto the calvarias. After 7 days, mice were sacrificed and tibia and calvarias were dissected. Samples were cleaned free of soft tissues and frozen in dry ice for RNA extraction. In another set of experiments, EC-CM-injected animals were sacrificed after 3 weeks, calvarias were dissected, mounted with 70% ethanol and analyzed by micro-CT.

### *In vivo* treatment with botulin toxin A

Eight‐week‐old C57BL/6 mice were subjected to botulin toxin A (Botox) injection as described previously^50^. Briefly, mice were anesthetized, then saline solution (20 mL) or Botox (2.0 unit/100 g) were injected into the left and the right hind limbs, respectively, into the quadriceps and the posterior compartment of the calf (targeting gastrocnemius, plantaris, and soleus). The behavioral response of each mouse was monitored on days 1, 3, 7, and weekly thereafter using whole body weight measurement and assessment of gait disability. After 21 days mice were sacrificed, then the hind limbs were removed, femurs and tibias were cleaned free of soft tissues and frozen in dry ice for RNA extraction.

### Hind limb tail suspension

Hind limb tail suspension was performed on 8‐week‐old C57BL/6 mice using the protocol described by Sakata *et al*.^51^. Briefly, a strip of elastic tape forming a half‐circle at the middle of the tail was applied to the ventral surface of the mice tail. A swivel attached to the half‐circle of the tape was fixed to an overhead wire, the height was adjusted to maintain the mice suspended at an approximately 30-degree angle. The swivel apparatus allowed animals to move freely into the cage using their forelimbs. After 21 days of suspension mice were euthanized by CO_2_ inhalation, hind limbs were removed and cleaned from soft tissues, then they were frozen in dry ice for the RNA extraction. An equal number of mice were maintained under normal cage conditions for 21 days as controls.

### Forced swim test

C57BL/6 mice subjected to tail suspension were allowed to swim in a 250‐L water‐filled tank (water depth = 20 cm), with water temperature kept at 31 °C. This test lasted 5 minutes and was repeated 3 times/day for 3 days/week during the entire period of unloading (3weeks)^52^. Control mice were released from tail suspension for the same time of swimming test.

### Statistics

Results are expressed as the mean ± SD of at least three independent *in vitro* experiments and at least 3 mice/group. Statistical analysis was performed by the Student’s *t*-test and one-way Repeated Measures Analysis of variance (RM ANOVA) according to the type of data sets. The statistical methods are indicated in the figure legends and the p-values are indicated in the figures. A p value < 0.05 was conventionally considered statistically significant.

## Additional Information

**How to cite this article**: Veeriah, V. *et al*. Interleukin-1β, lipocalin 2 and nitric oxide synthase 2 are mechano-responsive mediators of mouse and human endothelial cell-osteoblast crosstalk. *Sci. Rep.* **6**, 29880; doi: 10.1038/srep29880 (2016).

## Supplementary Material

Supplementary Information
