# Supplementary material for: Interleukin-1β, lipocalin 2 and nitric oxide synthase 2 are mechano-responsive mediators of mouse and human endothelial cell-osteoblast crosstalk
Source: Sci Rep. 2016 Jul 19;6:29880. doi: 10.1038/srep29880 (PMC4949438; doi:10.1038/srep29880)
Supplement: Supplementary Information [file srep29880-s1.pdf]

Supplementary Materials and Methods

Interleukin-1β, lipocalin 2 and nitric oxide synthase 2 are mechano-responsive mediators of mouse and human endothelial cell-osteoblast crosstalk

Vimal Veeriah[1,2], Angelo Zanniti[1], Riccardo Paone[1], Suvro Chatterjee[2], Nadia Rucci[1], Anna Teti[1], Mattia Capulli[1].

[1]Department of Biotechnological and Applied Clinical Sciences, University of L'Aquila, via Vetoio – Coppito 2, 67100 L'Aquila, Italy; [2]Vascular Biology Laboratory, Anna University K.B.Chandrashekar Research Centre, Chrompet. Chennai (Madras)-600 044, Chennai, India.

Materials

Dulbecco's-modified minimum essential medium (DMEM), fetal bovine serum (FBS), penicillin, streptomycin and trypsin were from Invitrogen (Carlsbad, CA). 1400W dihydrochloride (#W4262), hydroxyurea (#H8627) and cPTIO (#C221) were from Sigma-Aldrich (St. Louis, MO). Sterile plastic ware was from Falcon-Becton-Dickinson (Cowley, Oxford, UK) or Costar (Cambridge, MA). CultiSpher-G® microbeads (#D-G0001-OO) were from Percell Biolytica AB (Astorp, Sweden). BD Matrigel was obtained from BD Bioscience (#356234). The Trizol reagent, primers, and RT-PCR reagents were from Invitrogen. The Brilliant® SYBR® Green QPCR master mix was from Stratagene (La Jolla, CA). Enhanced ChemoLuminescence (ECL) kit and Hybond nitrocellulose were from Amersham Pharmacia Biotech (Little Chalfont, Bucks, UK). Anti-Lipocalin 2 (#AF1857), anti-mouse, anti-donkey and anti-goat IgG HRP-conjugated antibody (#HAF109) were obtained from R&D systems (Minneapolis, MN). Anti-VEGF (#K1703), PECAM (#sc1506), P65 (#C1914) and β-actin (#K2713) were from Santa Cruz Biotechnology, Inc. (Heidelberg, Germany). ELISA kit for mouse soluble IL-1β was from RoyBiotech, Inc (#ELM-IL-1beta-001). Recombinant RANK-L (#310-31) and M-CSF (#300-25) were from PEPROTECH. INK, Rocky Hill, NJ. All other reagents were from Sigma–Aldrich Co (St. Louis, MO).

Primary endothelial cell culture

Primary endothelial cells were isolated from mouse aorta as previously described[1]. Briefly, male CD1 mice were euthanized by CO_2 inhalation and abdomen skin was surgically removed and the thorax was opened. The abdominal aorta was cut to release the blood, and then a fine syringe was used to perfuse the vessel with 1000 U/ml heparin. The thoracic aorta was carefully dissected out from the aortic arch and immersed in 20% FBS-DMEM containing 1000 U/ml heparin. Then the fat and connective tissue were completely removed

using fine forceps under a stereoscopic microscope. The proximal portion of the aorta was inserted with 24-gauge cannula and ligated with a silk thread and the inside of the lumen was washed with serum free DMEM. The other side of the aorta was filled with type II collagenase solution and incubated for 45 minutes at 37ºC. Then the aorta was flushed with 5 ml of DMEM containing 20% FBS to release the endothelial cells, the supernatant was centrifuged at 1200 rpm for 5 minutes and the endothelial cells were collected. Finally, the endothelial cells were seeded on matrigel coated dishes containing culture medium [20% FBS, 100 U/ml penicillin–G, 100 µg/ml of streptomycin, 2 mM of L-glutamine, 1X sodium pyruvate, 25 mM HEPES (pH 7.0 – 7.6)] supplemented with 5 ng VEGF. After one week, endothelial cells were confluent and ready for the experimental procedures.

Human endothelial cell line

The human endothelial cell line, EA.hy926, was obtained from ATCC (CRL-2922) and was cultured in type 1 collagen-coated dish containing DMEM supplemented with 10% FBS, 1% penicillin/streptomycin in a humidified atmosphere of 5% CO_2 at 37°C.

Primary mouse osteoblast cultures

Calvarias from 7 day-old C57BL/6J wild type and LCN2 knockout mice were removed, cleaned thoroughly and digested three times with 1 mg/mL clostridium histolyticum type IV collagenase and 0.25% trypsin, for 20 minutes at 37°C, with gentle agitation. Cells from the second and third digestions were plated and grown in standard conditions, in DMEM containing 10% FBS and 1% penicillin/streptomycin. These cells expressed the osteoblast markers ALP, *Runx2*, *Osterix*, *Coll 1 chain-a*, and *osteocalcin*.

Primary human osteoblast cultures

Primary human osteoblasts were isolated from bone fragments collected from patients after traumatic bone fractures, under their informed consent and approval by the University of L'Aquila Ethics Board. The bone fragments were thoroughly cleaned from fat and soft tissues, chopped in small pieces and processed for osteoblast isolation as described above.

Total bone marrow cell culture

Bone marrow cells of the long bones of 7-day-old wild type and LCN2 KO mice (C57BL/6J background) were flushed out, diluted 1:1 in Hank's balanced salt solution and centrifuged at 400*g* for 30 minutes. Then cells were re-suspended in DMEM containing 10% FBS and plated in culture dishes at a density of 10^6 cells/cm^2. After 3 hours, cell cultures were rinsed to remove non-adherent cells and cultured with 1*g*-, 0.08*g* or 0.008*g*-EC-CM for 7 days.

Purified osteoclast cultures

Mouse primary osteoclast precursors were isolated from 7 day-old CD1 mice. The bone marrow from the bone cavity of the long bones was flushed out and diluted 1:1 in Hank's balanced salt solution, layered over Histopaque 1077 solution and centrifuged at 400g for 30 minutes. Cells were washed twice with Hank's solution, resuspended in DMEM supplemented with 10% FBS and plated in culture dishes at a density of 10^6 cells/cm^2. After 3 hours, cultures were rinsed to remove non-adherent cells and maintained for 7 days in 1*g*- or 0.008*g* EC-CM supplemented with 50ng/mL rhM-CSF.

MTT proliferation assay

Primary human or mouse osteoblasts (10000/well) were cultured in 96-well plates with 1*g*-, 0.08*g* or 0.008*g*-EC-CM. 48 hours later 20 µL of 5mg/mL of MTT solution was added to each well. After 3 hours, the solution was removed and 200 µL of DMSO was added and the color change was measured at 595 nm using a spectrophotometer.

Alkaline phosphatase activity assay

Primary human osteoblasts, or mouse osteoblasts from wild type or LCN2 KO mice, were cultured with 1*g*-, 0.08*g* or 0.008*g*-EC-CM for 48 hours. Cells were then fixed in 4% of paraformaldehyde for 15 minutes and washed twice with PBS. Analysis of ALP activity was evaluated cytochemically by the Sigma-Aldrich kit n. 85, according to the manufacturer's instruction. Quantitative analysis was performed by scanning densitometry using the Molecular Analyst software for the model 670 scanning densitometer (Bio-Rad Laboratories, Hercules, CA, USA) to obtain arbitrary density units.

Conventional and real-time RT-PCR

Total RNA was isolated from the endothelial cells, osteoblasts, calvarias and tibias using the TRIzol method. Two µg of RNA was reverse transcribed using the Moloney murine leukemia virus reverse transcriptase, and the equivalent of 0.1 µg was used for PCR reactions. Real-time PCR was performed employing the Brilliant SYBR Green QPCR master mix and conventional PCR by the green taq master mix. PCR conditions and primer pairs are listed in Supporting Table 2. Results were expressed as fold increase for real-time RT-PCR or shown by electrophoresis of PCR products in 2% agarose gel plus ethidium bromide for conventional RT-PCR. Results were normalized versus the housekeeping gene *Gapdh*.

Western blotting

For total protein extraction, osteoblasts or endothelial cells were lysed in RIPA buffer (50 mM Tris–HCl, pH 7.5, 150 mM NaCl, 1% Nonidet P-40, 0.5% sodium deoxycholate, 0.1% SDS) containing protease inhibitors, then proteins were resolved by 10% SDS–PAGE and transferred to nitrocellulose membranes. Blots were probed with the primary antibody for 1

hour at room temperature, washed and incubated with the appropriate HRP-conjugated secondary antibody for 1 hour at room temperature. Protein bands were revealed by enhanced ECL.

Mineralization assay

Mouse primary osteoblasts were cultured with 1*g*- and 0.008*g*-EC-CM along with the supplementation of 10 mM β-glycerophosphate and 50 mg/ml of ascorbic acid for 21 days. Analysis of mineralization was performed by the Von Kossa staining.

Von Kossa staining

Osteoblast cells grown in 48 wells/plate were fixed in 4% of paraformaldehyde for 15 minutes and washed twice with distilled water. Cells were then incubated under UV light for 1 hour with 300 µL of 5% silver nitrate ($AgNO_3$). The solution was removed and dishes washed twice with distilled water. Then 300 µL of 5% sodium thiosulfate ($Na_2S_2O_3$) was added and dishes incubated for 2 minutes. The solution was removed, dishes were washed with distilled water and images were taken.

Immunofluorescence

Mouse primary osteoblasts or primary endothelial cells on microbeads were fixed with 4% paraformaldehyde and incubated for 1 hour at room temperature with specific primary antibodies followed by Fluorescein Isothiocyanate (FITC)- or tetramethyl rhodamine isothiocyanate (TRITC)-conjugated secondary antibody. To detect nuclei, cells were stained with 4,6diamidino-2-phenylindole (DAPI) or propidium iodide (PI). Images were taken by conventional epifluorescence (Axioplan; CarlZeiss, Inc., Thornwood, NY, USA) or confocal (FluoViewIX81FVBF; Olympus, Tokio, Japan) microscope. For fluorescence microscopy,

2.5× NA 0.075, 10× NA 0.30, 20× NA 0.5, and 40× NA 0.75 Plan-Neofluar objective lenses were used. Images were captured with a camera (AxioCam MRC5; Carl Zeiss, Inc.) using the AxioVs 40 version 4.7.1.0 software (Carl Zeiss, Inc.). For confocal microscopy, 10× NA0.30 and 40× NA0.85UPlan-Apochromator60× NA 1.4 oil Plan-Apochromat objective lenses were used. Images were captured using FluoView 500 software (Olympus).

TRAcP activity assay

Osteoclasts, total bone cells or whole calvarial bones were treated with 1*g*-, 0.08*g* or 0.008*g* EC-CM. After 7 days, cells were fixed in 4% paraformaldehyde for 15 min and extensively washed with the PBS. TRAcP activity was analyzed cytochemically using the Sigma-Aldrich kit no 386 according to the manufacturer's instruction.

Micro-CT analysis

Calvaria images were acquired in a SkyScan 1174 micro-CT scanner, with a voxel size of 6 µm (X-ray voltage 50kV). The Skyscan Nrecon software was used for image reconstruction by employing a modified Feldkamp algorithm. Beam hardening correction and Fourier transform–based ring artefact reduction were applied to the reconstructed images. Area of the bone fraction was calculated for the calvarial bone selected regions of interest[2] using the Image J software.

Bone histomorphometry of calvarial bones

Mice were subjected to subcutaneous injection of 1*g*-, or 0.008*g*-EC-CM in the area above the calvarial bones, and to intraperitoneal double injection of calcein, 7 and 2 days before sacrifice, performed after 7 days from EC-CM injection. Calvarial bones were harvested, fixed in 4% paraformaldehyde, dehydrated in ethanol and processed for methacrylate

embedding without decalcification. Histomorphometric measurements were carried out on 5-μm thick coronal sections, with an interactive image analysis system (IAS 2000; Delta Sistemi, Rome, Italy)[3] and with the suggested nomenclature[4]. Osteoblast number/bone perimeter was quantified after staining the sections with toluidine blue, while dynamic assessment of the mineral apposition rate was calculated through the calcein labeling. Bone formation rate was computed according to the following formula: mineral apposition rate × mineralized surface/bone area[4].

Supporting References

1. Kobayashi, M., Inoue, K., Warabi, E., Minami, T., & Kodama, T. A simple method of isolating mouse aortic endothelial cells. *J. Atheroscler. Thromb.* **2**, 138–142 (2005).
2. Bouxsein, M.L., Boyd, S.K., Christiansen, B.A., Guldberg, R.E., Jepsen, K.J., & Müller, R. Guidelines for assessment of bone microstructure in rodents using micro–computed tomography. J. Bone Miner. Res. **25**,1468–1486 (2010).
3. Rucci, N., et al. The glycosaminoglycan-binding domain of PRELP acts as a cell type-specific NF-kappaB inhibitor that impairs osteoclastogenesis. *J. Cell Biol.* **187**, 669–683 (2009).
4. Dempster, D.W., et al. Standardized nomenclature, symbols, and units for bone histomorphometry: a 2012 update of the report of the ASBMR Histomorphometry Nomenclature Committee. *J. Bone Miner. Res.* **28**, 2–17 (2013).

Supplementary Table S1. Animal methods and ARRIVE compliant.

Ethical statement	Methods involving animal care conformed national and international laws and policies (EEC Council Directive 86/609, OJ L 358, 1, Dec. 12, 1987; Italian legislative Decree 116/92, Gazzetta Ufficiale della Repubblica Italiana n. 40, Feb. 18, 1992; Guide for the Care and Use of Laboratory Animals: Eighth Edition, ISBN-10: 0-309-15396-4), and were approved by the Institutional Review Board of the University of L'Aquila.
Study design	Isolation of primary cells<br>a. Experimental and control groups derived by the 5 mice<br>b. Samples obtained from aorta and calvarias immediately used for cell cultures<br>c. Experimental unit: animals from the same littermate.<br>Injection of 0.008g-EC-CM onto mouse calvarias and into mouse tibia<br>a. Experimental groups 3 mice, control group 3 mice<br>b. Samples obtained from mice from the second group of experiments were stored and will be used for other projects, according to the 3Rs rule (reduce)<br>c. Experimental unit: group of animals in different cages<br>Induction of bone unloading in vivo<br>a. Experimental groups 3-5 mice, control group 3-5 mice<br>b. Samples were obtained from a set of in vivo experiments performed for another project, according to the 3Rs (refine)<br>c. Experimental unit: groups of animals in different cages
Experimental procedures	Anesthesia: Ketamine/xylazine cocktail, 87.5 mg/kg Ketamine, 12.5 mg/kg Xylazine, ophthalmic ointment was applied to both eyes to prevent desiccation.<br>To recover from anesthesia mice were placed in warm, clean, dry, quiet environment away from other animals. Commercially-available surgical heating pad were used to warm up

	animals. Bedding material was replaced with toweling material to avoid bedding to stick to eyes or be inhaled while animals are recovering from anesthesia.<br>All the operation on animals were done in the morning and finished before noon and animals monitored for further 5 hours.<br>Euthanasia: At the end of the experiments, mice (both genders) were euthanized by CO2 inhalation
Experimental animals	Mice used for primary cultures were from the C57BL6/j strain, both genders, age 7 days, weight 5 ± 1gr.<br>Genotype: WT and LCN2 KO<br>Mice used for in vivo experiments were from the C57BL6/j strain, both genders, age 2 months, weight 15 ± 3gr.<br>Genotype: WT<br>Immunocompetent
Housing and husbandry	Animal facility: standard<br>Temperature 20-24°C<br>Diet: access to food and water ad libitum, normal diet (Mucedola code: 3KE25)<br>Dark/light cycle: 12/12hours<br>Humidity: 60% ± 5<br>Cage: plastic<br>Cage companions, 3 adults/cage, gender are not mixed<br>Bedding material: hi adsorbing power, without dust. Changed every week.<br>Environmental enrichment was done with sterile material.
Sample size	Small groups of 3 animals/group were designed for the experiments where 0.008g-EC-CM, being the differences expected big enough to reduce to minimum the number of replicates.<br>Unloading experiments were conducted on larger sized groups.

	The sample size was calculated for both experiments using dedicated software (sigmaplot), basing on the expected differences.<br>The relevance of the analyzed pathway allowed us to obtain statistic significant results with relatively little sample sizes.
Allocating animals to experimental groups	Animals were assigned to groups after randomization.
Experimental outcomes	To test the activation of the identified pathway in in vitro primary cells and in vivo, and to test its relevance in in vivo models of disuse osteoporosis due to mechanical unloading.
Statistical	Statistical analysis was performed by the Student's *t*-test and one-way Repeated Measures Analysis of variance (RM ANOVA) according to the type of data sets. The statistical methods are indicated in the figure legends and the p-values are indicated in the figures. A p value<0.05 was conventionally considered statistically significant.

Supplementary Table S2. Primer pairs used throughout the study. PCR conditions were 94°C for 30s, 60°C for 30s, and 72°C for 30s, replicated for 40 cycles for real-time RT-PCR and 27 cycles for semi-quantitative RT-PCR.

Gene	Forward Primer	Reverse Primer
Alp	5’-CCAGCAGGTTTCTCTCTTGG-3’	5’-CTGGGAGTCTCATCCTGAGC-3’
Coll-1	5’-CCGTGCTTCTCAGAACATCA-3’	5’-GAGCAGCCATCGACTAGGAC-3’
Cox2	5’-AGAAGGAAATGGCTGCAGAA-3’	5’-GCTCGGCTTCCAGTATTGAG-3’
Cyclin-1	5’-AGCGCTGTTTTTGTTGTGTG-3’	5’-CCTTCCGGTGTGAAACATCT-3’
Gapdh	5’-TGGCAAAGTGGAGATTGTTGC-3’	5’-AAGATGGTGATGGGCTTCCCG-3’
IL17	5’-GCCCTCCACAATGAAAAGAA-3’	5’-TTTCACCCCATTCAGAGGAG-3’
IL1-β	5’-ATGGCAACTGTTCCTGAACTCAAGT-3’	5’-CAGGACAGGTATAGATTCTTTCCTT-3’
Lcn 2	5’-CCAGTTCGCCATGGTATTTT-3’	5’-CACACTCACCACCCATTCAG-3’
Nos2	5’-CACCTTGGAGTTCACCCAGT-3’	5’-AACCACTCGTACTTGGGATGC-3’
Osterix	5’-TGCTTCCCAATCCTATTTGC-3’	5’-AGAATCCCTTTCCCTCTCCA-3’
Opg	5’-AAAGCACCCTGTAGAAAACA-3’	5’-CCGTTTTATCCTCTCTACACTC-3’
RankL	5’-CCAAGATCTCTAACATGACG-3’	5’-CACCATCAGCTGAAGATAGT-3’
Runx2	5’-AACCCACGGCCCTCCCTGAACTCT-3’	5’-ACTGGCGGGGTGTAGGTAAAGGTG-3’
TNFα	5’-GCAGGTCTACTTTGGAGTCATTGC-3’	5’-TCCCTTTGCAGAACTCAGGAATGG-3’

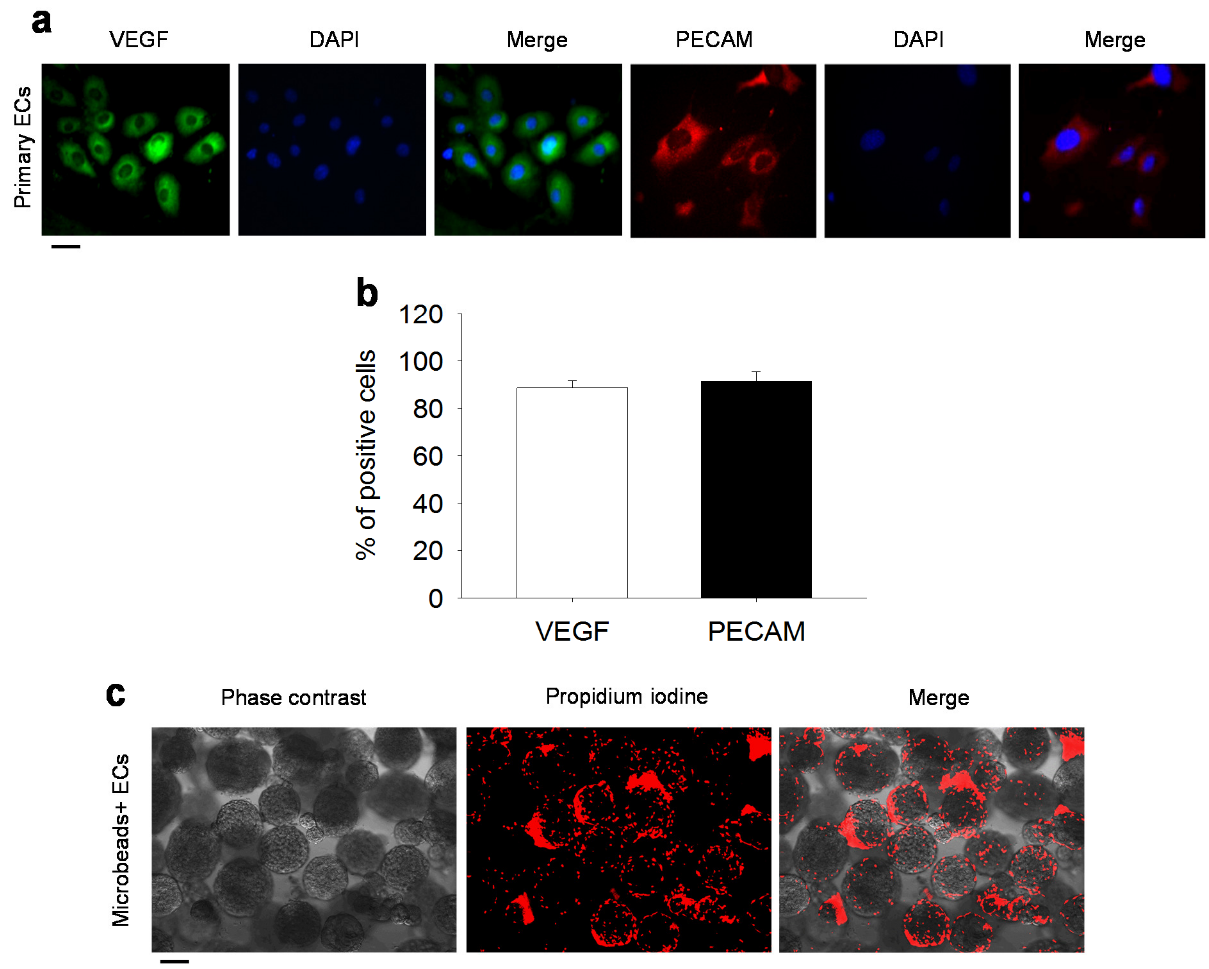

Supplementary Fig. S1. Primary mouse endothelial cell characterization. (a) Primary endothelial cells (ECs) were isolated from 7 week-old CD1 mouse aorta and characterized by immunofluorescence using antibodies against the endothelial specific markers, VEGF and PECAM. Nuclei were stained with DAPI. **(b)** VEGF and PECAM positive cells per field were counted and converted in percent of total cells. **(c)** Mouse primary endothelial cells were loaded onto microbeads for microgravity experiment. Phase contrast microscopy (left panel), propidium iodide staining of cell nuclei (red) (middle panel) and merged images (right panel). Images are representative and data are the mean±SD of 3 independent cell preparations (Student's *t* test). Bar = (A) 20 µm; (C) 60 µm.

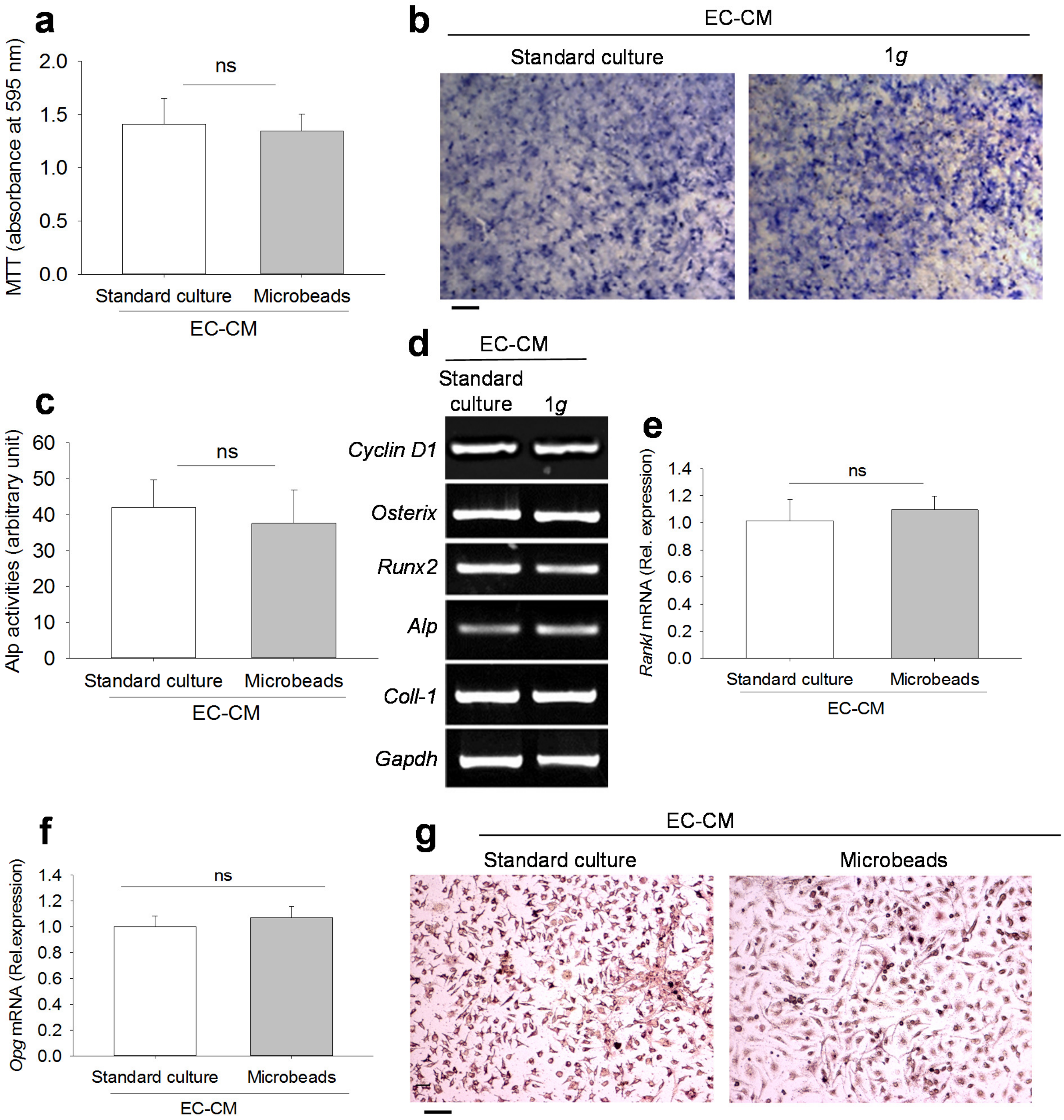

Supplementary Fig. S2. Control culture conditions. Mouse calvaria osteoblasts were cultured in EC-CM obtained from mouse endothelial cells cultured in standard adherent monolayer conditions or on microbeads incubated in non-adherent Petri dishes, representing our 1*g* endothelial cell cultures. **(a)** MTT proliferation assay. **(b)** Representative images of ALP cytochemical staining. **(c)** Densitometric quantification of ALP. **(d)** RT-PCR analysis of the indicated genes normalized versus *Gapdh.* **(e)** Real time RT-PCR analysis of *Rankl*

normalized versus *Gapdh*. **(f)** Real time RT-PCR analysis of *Opg* normalized versus *Gapdh*. **(G)** Total bone marrow cells were isolated from the long bones of 7 day-old CD1 mice and cultured with EC-CM obtained from mouse endothelial cells cultured in standard adherent monolayer conditions and on microbeads incubated in non-adherent Petri dishes, representing our 1*g* cultures conditions. Representative images of TRAcP staining. Images are representative and data are the mean±SD of 3 independent experiments (Student's *t* test; ns = not significant). Bar = 40 μm. All gels have been run under the same experimental conditions.

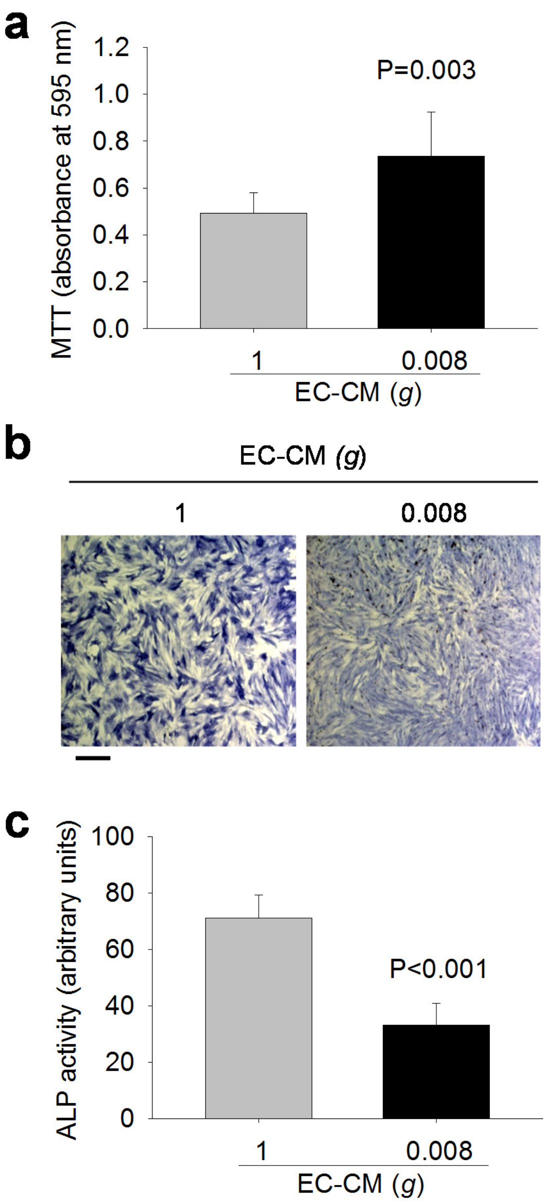

Supplementary Fig. S3. Effect of 0.008*g*-EC-CM on human osteoblasts. Human endothelial cells (EA.hy926 cells) were subjected for 96 hours to 1*g* and 0.008*g*, then EC-CM were collected and used to treat primary osteoblasts isolated from healthy human bone samples. **(a)** MTT proliferation assay. **(b)** Representative images of ALP cytochemical staining. **(c)** Densitometric quantification of ALP. Images are representative and data are the mean±SD of 3 independent experiments (Student's *t* test). Bar = 40 µm.

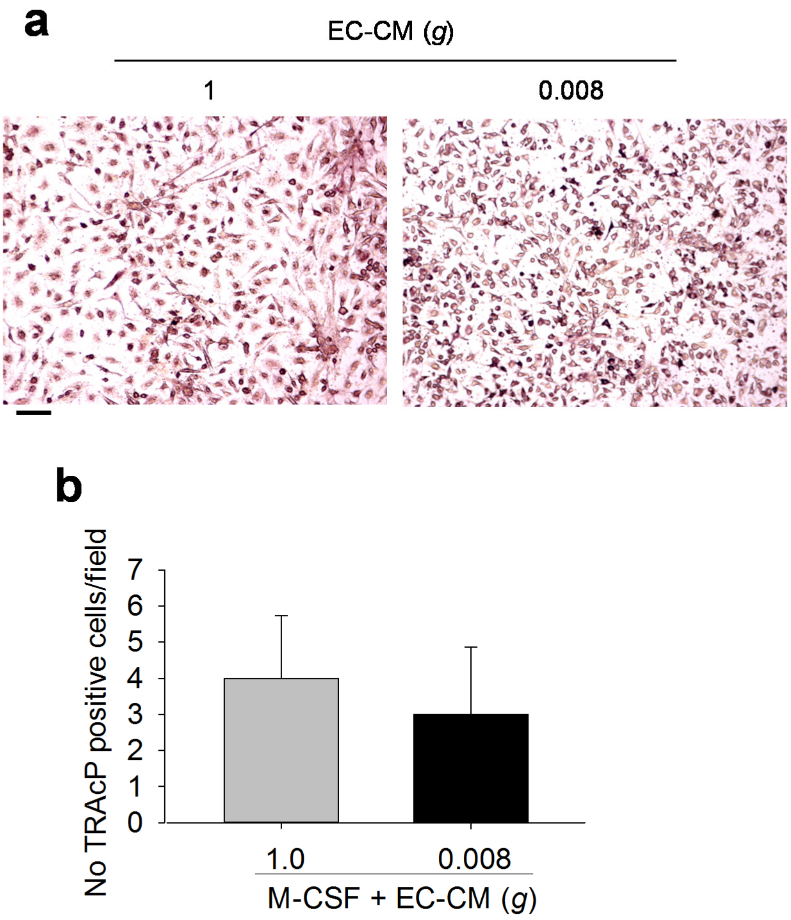

Supplementary Fig. S4. Lack of effect of 0.008*g*-EC-CM on osteoclastogenesis in purified osteoclast precursor cultures. Bone marrow mononuclear cells were purified from the long bones of 7-day old CD1 mice by centrifugation on Ficoll gradient and treated with 1*g*- or 0.008*g*-EC-CM along with M-CSF to trigger expression of the RANKL receptor, RANK. **(a)** Images of TRAcP staining. **(b)** Number of TRAcP positive multinucleated cells. Images are representative and data are the mean±SD of 3 independent experiments (Student's *t* test). Bar = 40 µm.

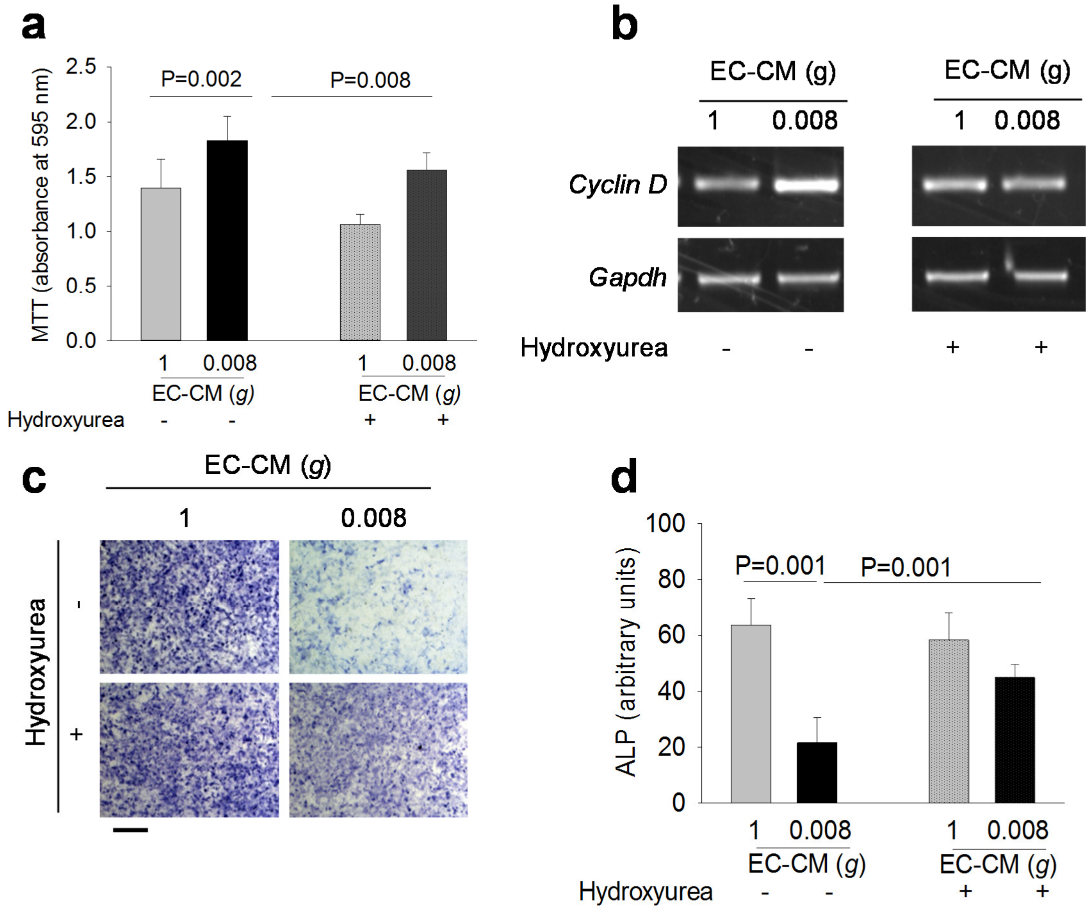

Supplementary Fig. S5. Effect of hydroxyurea on endothelial cell-osteoblast crosstalk. Mouse primary osteoblasts were treated with 1*g*- or 0.008*g*-EC-CM in the absence or presence of the proliferation inhibitor, hydroxyurea. **(a)** MTT proliferation assay. **(b)** RT-PCR analysis of *Cyclin D1* normalized versus *Gapdh*. **(c)** Representative images of ALP cytochemical staining. **(d)** Densitometric analysis of ALP staining. Images are representative and data are the mean±SD of 3 independent experiments (Student's t test). Bar = 100 µm. All gels have been run under the same experimental conditions.

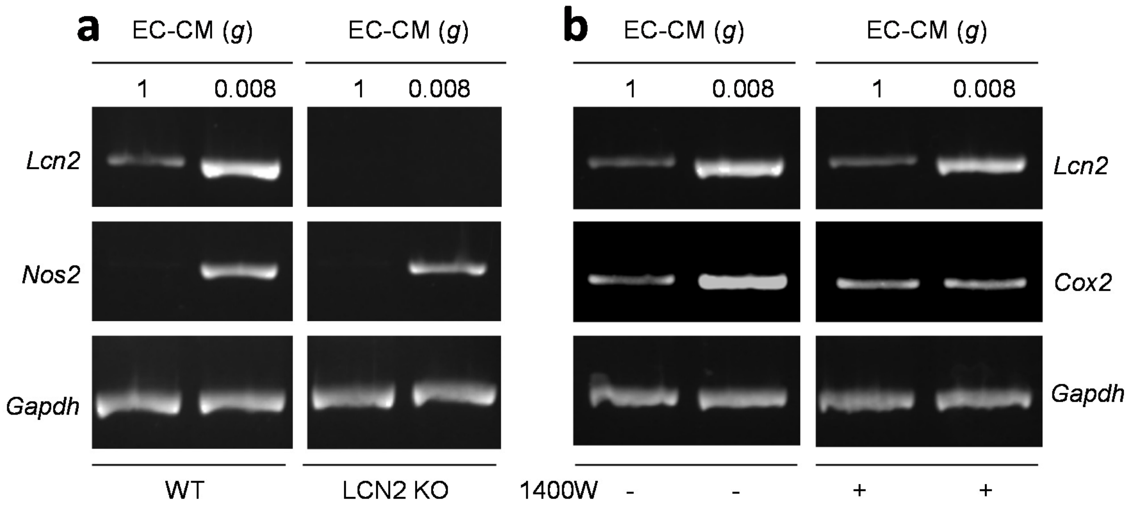

Supplementary Fig. S6. LCN2 and NOS2 signaling in osteoblasts are independent to each other. (a) Osteoblasts were isolated from wild type (WT) and LCN2 KO mice and treated with 1*g*- and 0.008*g*-EC-CM. RT-PCR of *Lcn2* and *Nos2* normalized versus *Gapdh*. **(b)** Osteoblasts were treated with 1*g*- and 0.008*g*-EC-CM with or without the NOS2 inhibitor 1400W. RT-PCR of *Lcn2* and *Cox2* normalized versus *Gapdh.* Images are representative of 3 independent experiments. All gels have been run under the same experimental conditions.

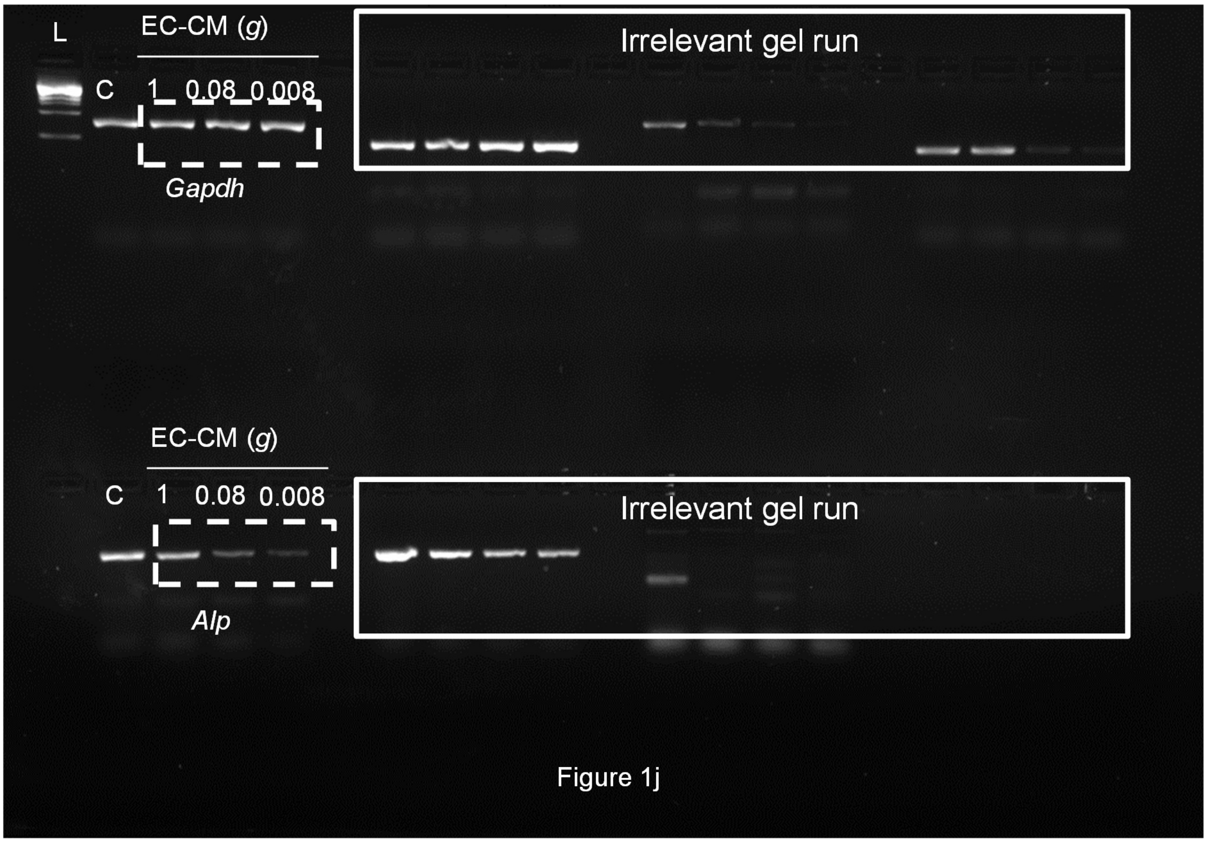

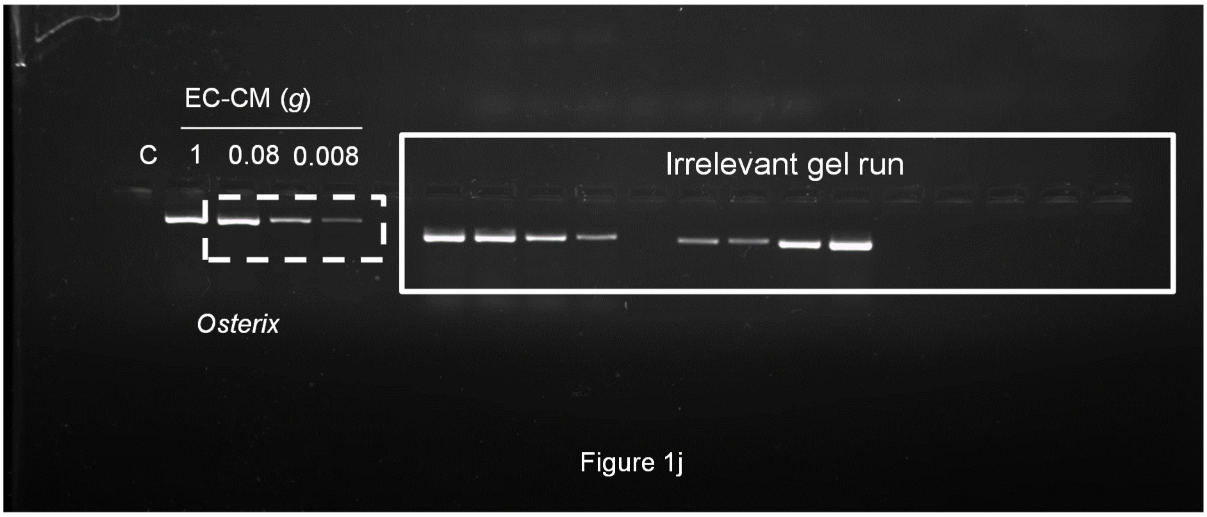

Supplementary Fig. S7. Uncropped PCR gel pictures correspondent to main Fig. 1j. L, base pairs marker; C, DMEM treatment; EC-CM; endothelial cell-conditioned media treatment; 1, 1*g*, 0.08, 0.08*g*, 0.008, 0.008*g* microgravity. PCR bands inside dashed-line boxes are displayed in the main figure. PCR bands inside the solid-line boxes are irrelevant to the article.

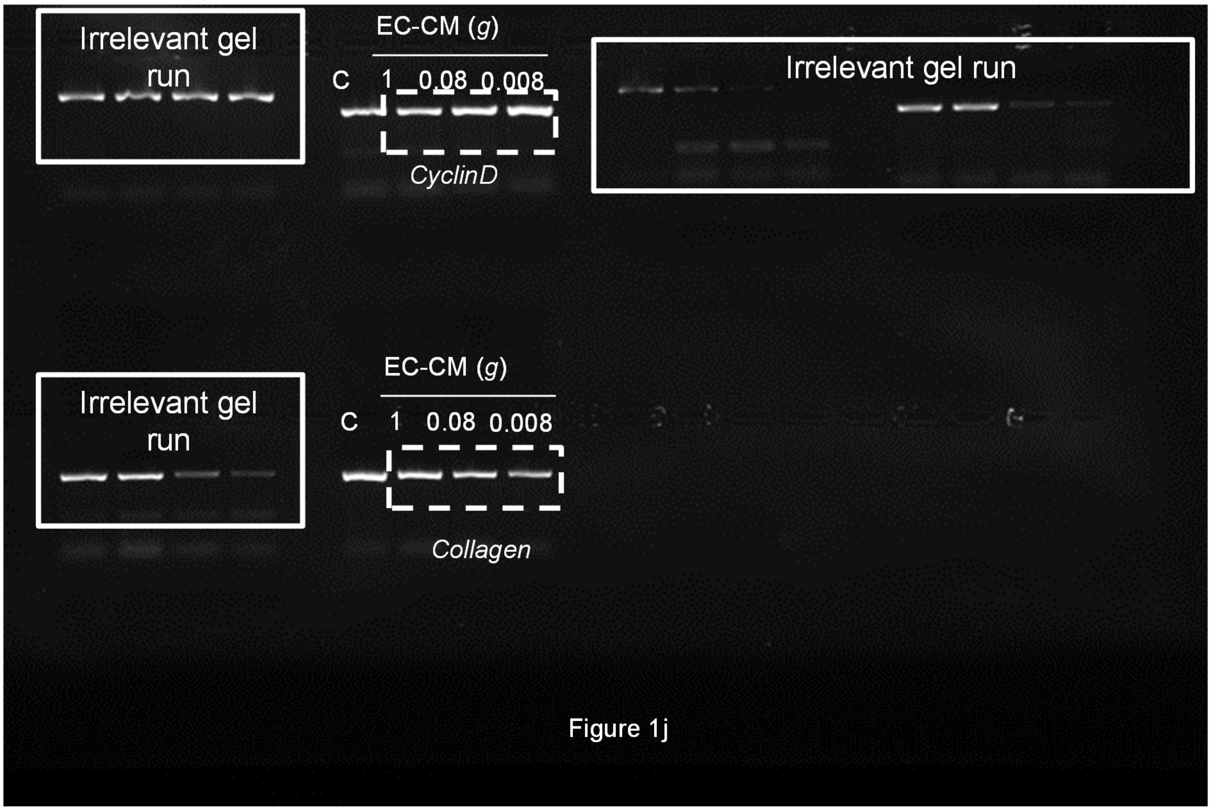

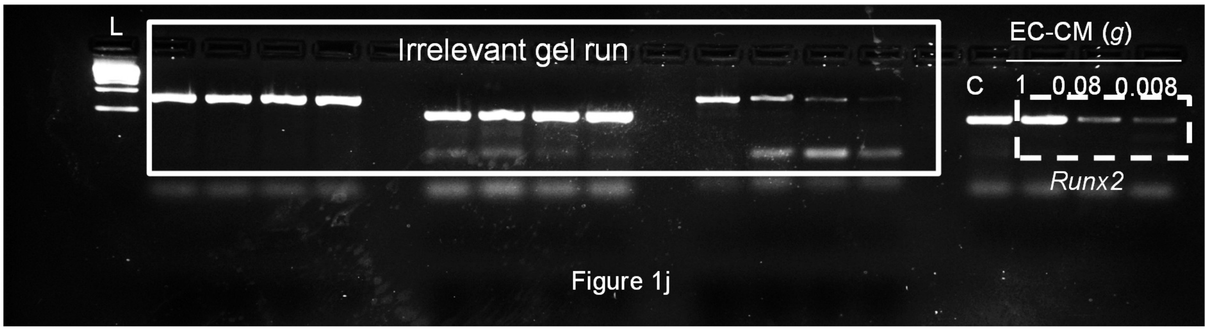

Supplementary Fig. S7 continued. Uncropped PCR gel pictures correspondent to main Fig. 1j. L, base pairs marker; C, DMEM treatment; EC-CM, endothelial cell-conditioned media treatment; 1, 1*g*; 0.08, 0.08*g;* 0.008,0.008*g*. PCR bands inside dashed-line boxes are displayed in the main figure. PCR bands inside the solid-line boxes are irrelevant to the article.

a

b

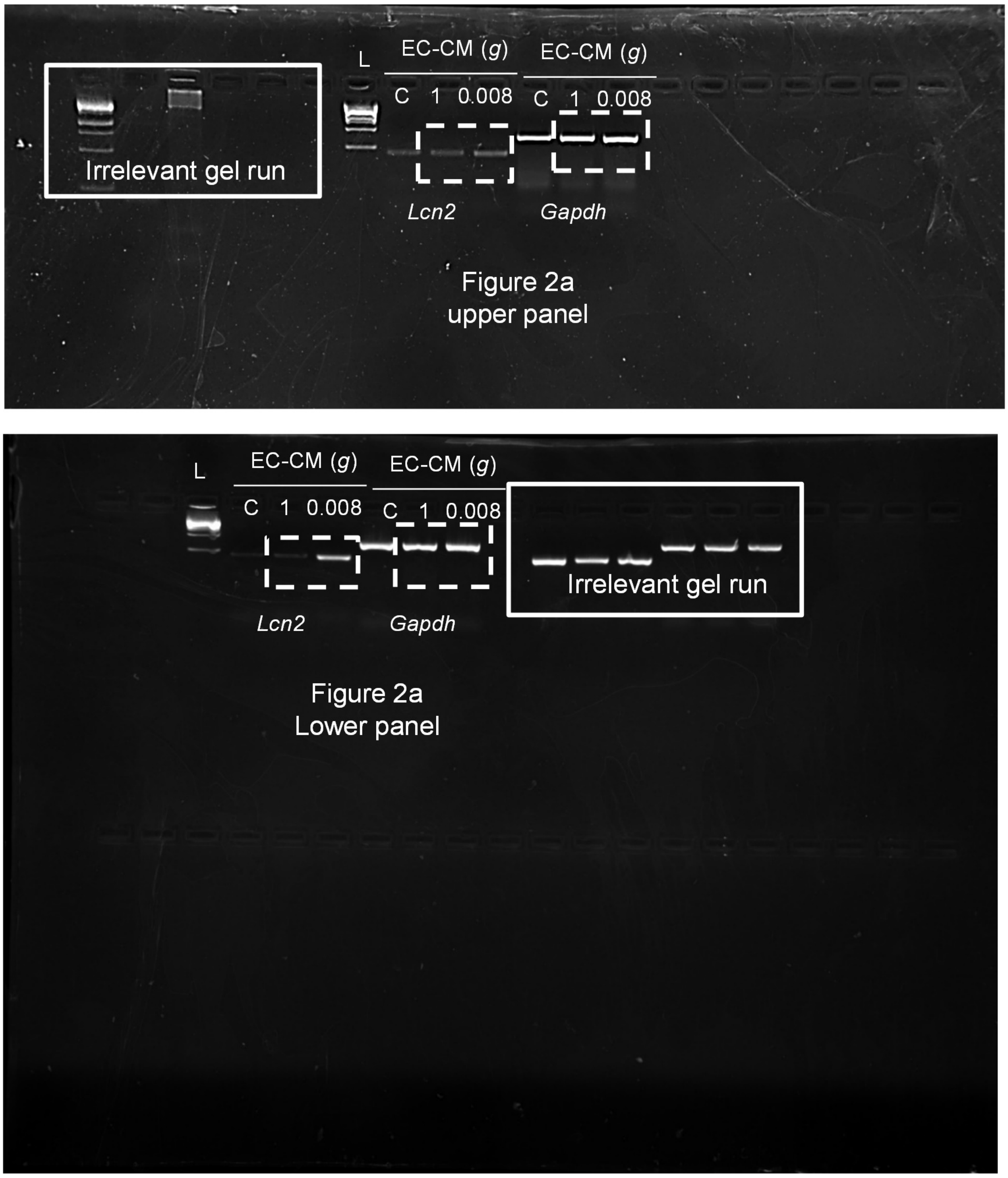

Supplementary Fig. S8. Uncropped PCR gel pictures correspondent to main Fig. 2a. L, base pairs marker; C, DMEM treatment; EC-CM, endothelial cell-conditioned media treatment; 1, 1*g*; 0.08, 0.08*g;* 0.008,0.008*g*. PCR bands inside dashed-line boxes are displayed in the main figure. PCR bands inside the solid-line boxes are irrelevant to the article.

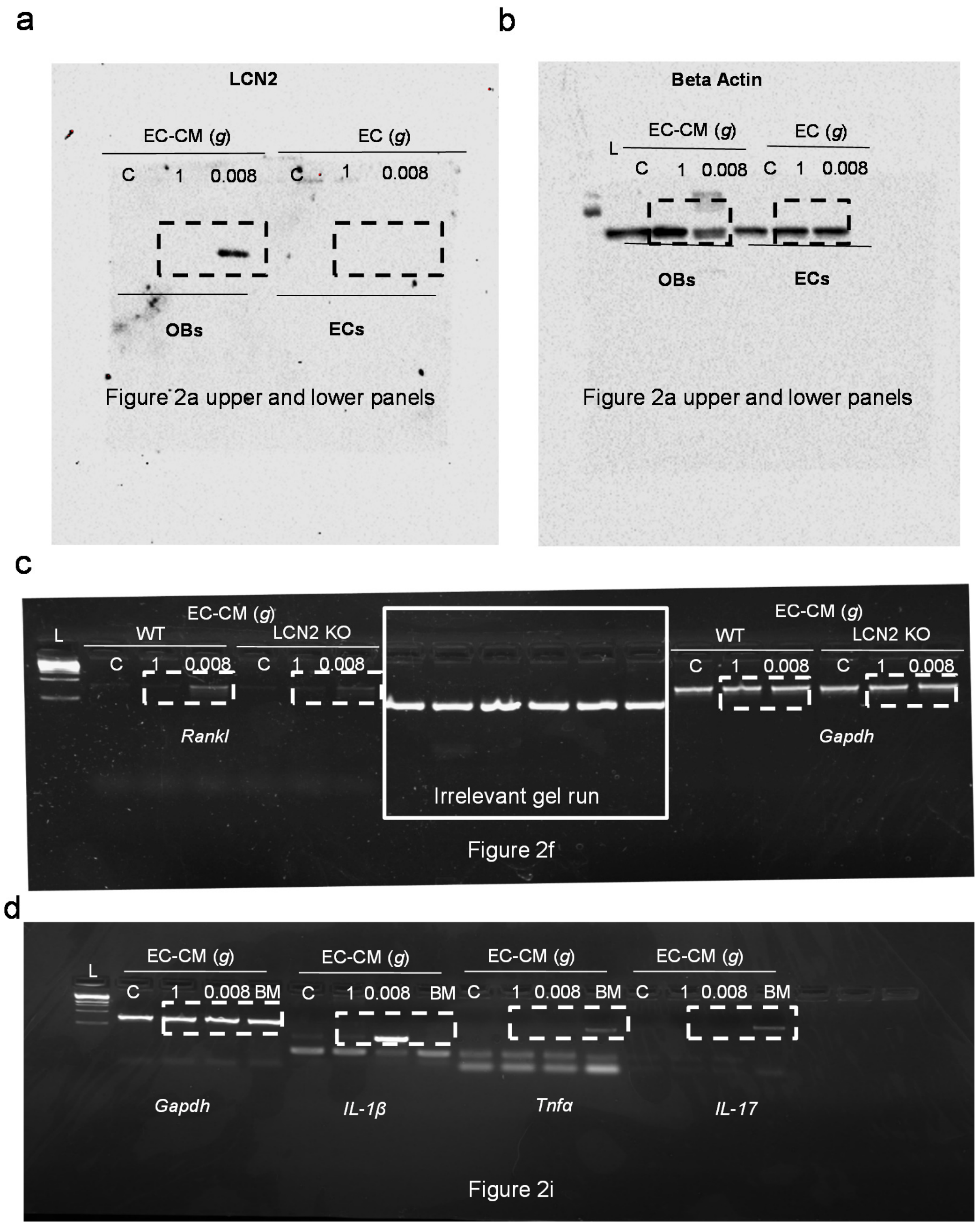

Supplementary Fig. S9. Uncropped PCR and western-blot gel pictures correspondent to the main Fig. 2a, f and i. L, base pairs marker; C, DMEM treatment; EC-CM, endothelial cell-conditioned media treatment; 1, 1*g*; 0.08, 0.08*g;* 0.008,0.008*g*. PCR and Western-blot bands inside dashed-line boxes are displayed in the main figure. PCR bands inside the solid-line boxes are irrelevant to the article.

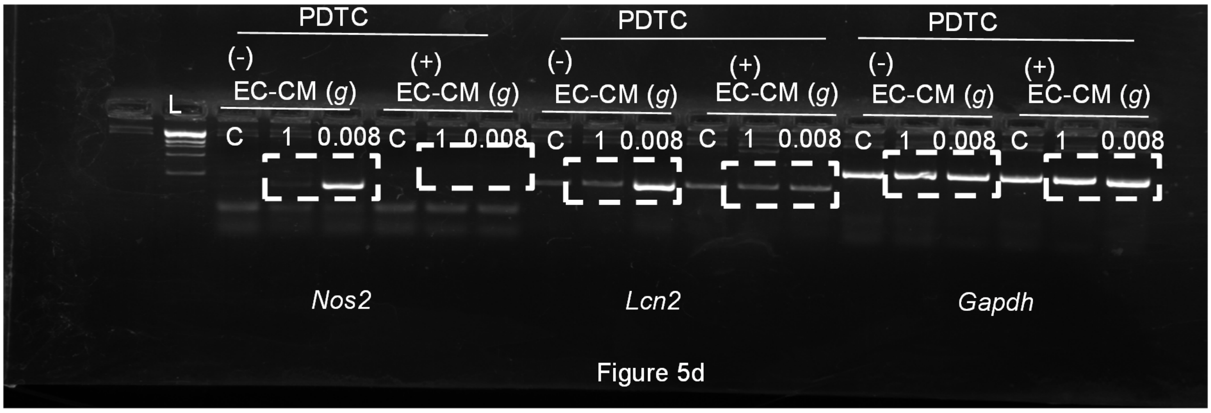

Supplementary Fig. S10. Uncropped PCR gel pictures correspondent to main Fig. 5d. L, base pairs marker; C, DMEM treatment; EC-CM, endothelial cell-conditioned media treatment; 1, 1*g*; 0.08, 0.08*g;* 0.008,0.008*g*. PCR bands inside dashed-line boxes are displayed in the main figure. PCR bands inside the solid-line boxes are irrelevant to the article.

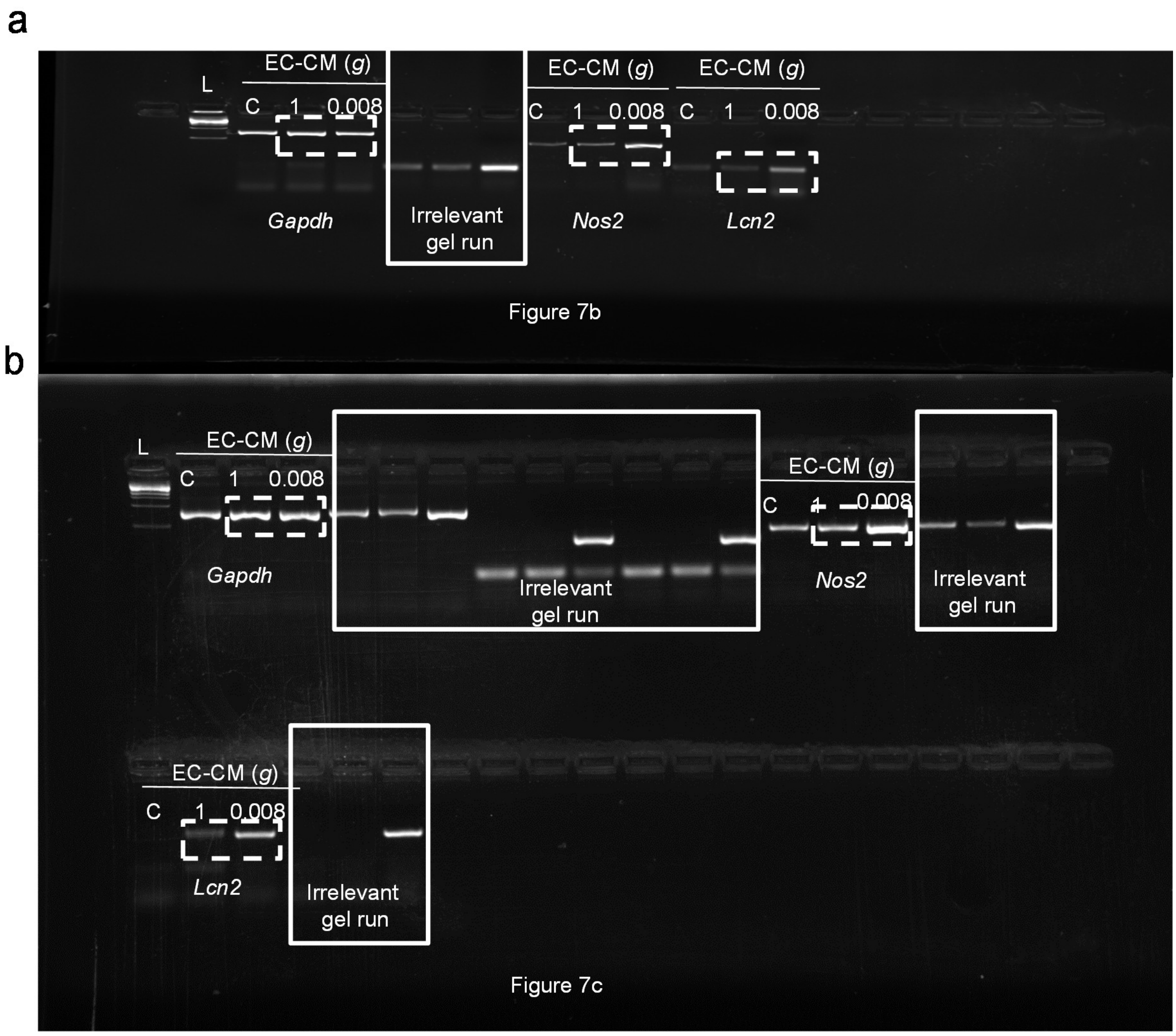

Supplementary Fig. S11. Uncropped PCR gel pictures correspondent to main Fig. 7b and c. L, base pairs marker; C, DMEM treatment; EC-CM, endothelial cell-conditioned media treatment; 1, 1*g*; 0.08, 0.08*g;* 0.008,0.008*g*. PCR bands inside dashed-line boxes are displayed in the main figure. PCR bands inside the solid-line boxes are irrelevant to the article.
